# Advances in neurotensin receptor 1-targeted molecular probes for tumor molecular imaging and therapy

**DOI:** 10.3389/fonc.2026.1817036

**Published:** 2026-04-24

**Authors:** Chengkuan Liu, Jiang Fu, Shengjie Tang, Haiyang Hu, Haiyang Guo, Tao Liu, Long Wen, Yang Yang, Yunlong Yang, Shuxuan Chen, Li Yu, Haining Zhou

**Affiliations:** 1 Department of Thoracic Surgery, Suining Central Hospital, Suining, Sichuan, China; 2 Department of Thoracic Surgery, Affiliated Hospital of Southwest Medical University, Luzhou, China; 3 Institute of Surgery, Graduate School, North Sichuan Medical College, Nanchong, China; 4 Institute of Surgery, Graduate School, Chengdu University of Traditional Chinese Medicine, Chengdu, China; 5 Department of Neurology, Affiliated Hospital of Southwest Medical University, Luzhou, China; 6 Department of Physical Examination, Suining Central Hospital, Suining, Sichuan, China

**Keywords:** fluorescence imaging, molecular probes, neurotensin receptor 1, PET imaging, theranostics

## Abstract

Neurotensin receptor 1 (NTS1) is a high-affinity G protein-coupled receptor (GPCR) for the endogenous peptide neurotensin (NT). NTS1 is overexpressed across multiple malignancies and is an important target for precision theranostics. In recent years, NTS1-targeted molecular probes have advanced considerably, improving the sensitivity and specificity of molecular imaging, including fluorescence imaging, positron emission tomography (PET), single-photon emission computed tomography (SPECT), and multimodal PET/fluorescence imaging, while also showing promise for response assessment and therapeutic decision-making. In parallel, theranostic strategies that integrate molecular imaging with targeted therapy enable a closed-loop workflow for patient stratification, individualized treatment, and response monitoring. In this review, we summarize recent advances in NTS1-targeted molecular probes, with particular emphasis on their clinical applications in tumor molecular imaging, radioligand therapy, and integrated diagnostic and therapeutic management. We integrate evidence from preclinical models and early human studies and outline the main challenges that currently limit their clinical translation. Overall, this review aims to provide a useful reference for the continued development of NTS1-targeted molecular probes in precision oncology.

## 1 Introduction

Cancer remains a major global public health burden, with consistently high incidence and mortality worldwide (1, 2). Although conventional approaches, including tissue biopsy, surgical resection, chemotherapy, and radiotherapy, have prolonged patient survival, they still face substantial challenges. These include limited sensitivity for early detection, insufficient treatment specificity, significant systemic toxicity, and frequent recurrence and metastasis. More precise tools are therefore urgently needed to address these limitations. In recent years, with rapid advances in molecular biology and medical imaging, molecular probes have enabled visualization at the molecular level and targeted drug delivery through the specific recognition of tumor biomarkers, thereby offering a new approach to precision diagnosis and therapy (3–5). Different types of molecular imaging probes have distinct clinical applications and advantages in cancer diagnosis and treatment. For diagnosis, fluorescent probes generate optical signals, providing high spatial resolution and real-time imaging. They are mainly used for intraoperative navigation, such as identifying small residual lesions and lymph node metastases, image-guided minimally invasive procedures, including endoscopic or biopsy localization, and the rapid detection of superficial tumors. However, their tissue penetration is limited, typically to less than 1 cm, which restricts their application for whole-body evaluation (6–8). Nuclear probes, which detect γ-rays or positrons emitted by radionuclides such as ^68^Ga and ^99m^Tc, provide whole-body quantitative functional and metabolic imaging via SPECT/PET and are mainly used for precise tumor staging, dynamic therapy assessment, surveillance of recurrence/metastasis, and therapeutic target delineation (9–11). Their strengths include strong tissue penetration and quantification capability, but they offer relatively low spatial resolution and do not support real-time imaging. In therapy, current theranostic strategies conjugate therapeutic radionuclides (e.g., ^177^Lu, ^225^Ac) to targeting ligands, delivering selective internal radiotherapy to tumors with high target expression and thereby enhancing efficacy while reducing systemic toxicity (12).

**Scheme 1:**
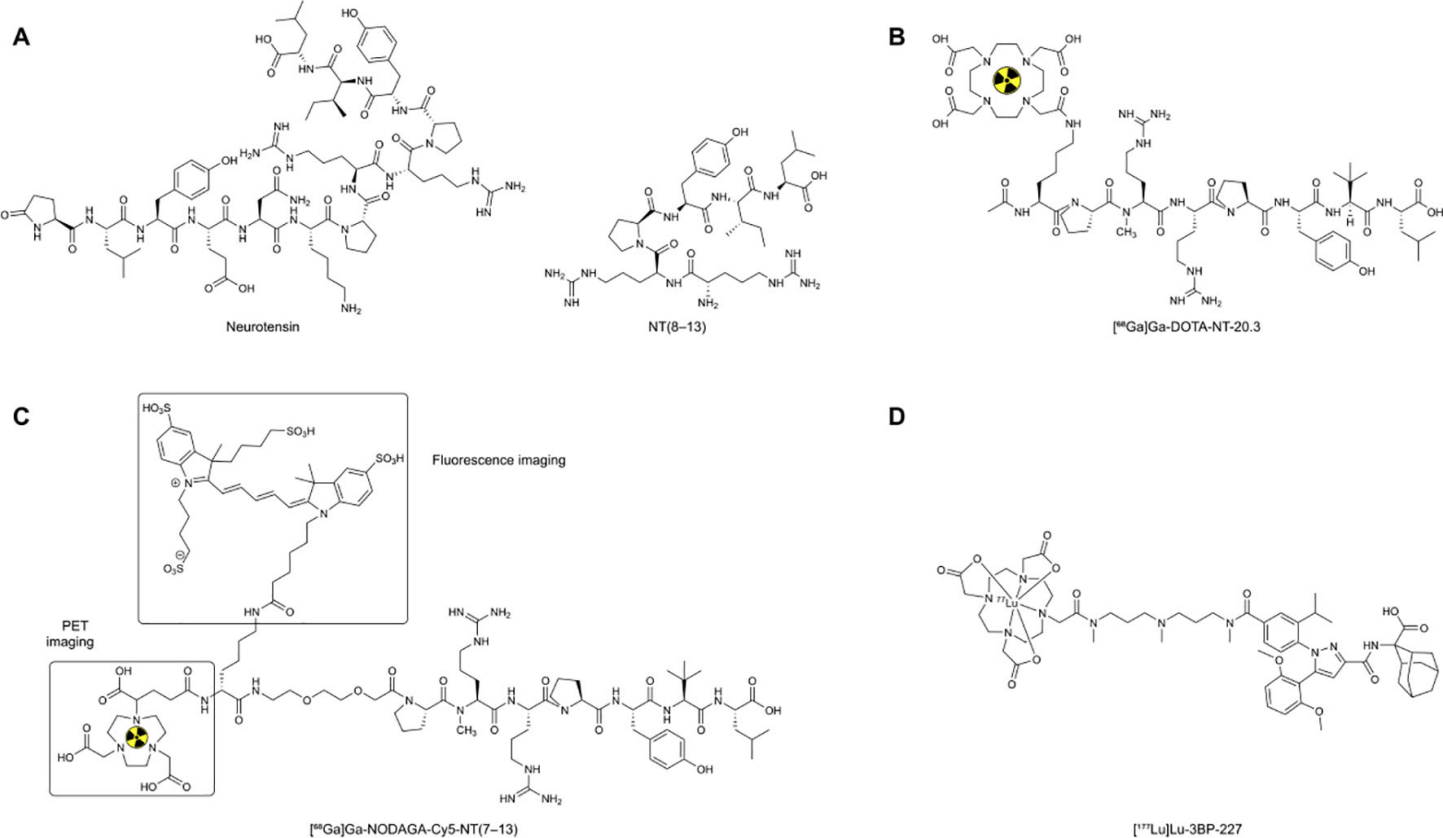
Representative chemical structures of major NTS1-targeted ligands7 and probes discussed in this review. **(A)** Neurotensin (NT, 1–13) and its C-terminal active fragment NT(8–13), which represents the core pharmacophore for peptide-based NTS1-targeted ligands. **(B)** [^68^Ga]Ga-DOTA-NT-20.3, shown as a representative peptide-based PET imaging probe. **(C)** [^68^Ga]Ga-NODAGA-Lys(Cy5)-AEEAc-[Me-Arg8,Tle12]-NT(7–13), shown as a representative dual-modality probe for PET imaging and fluorescence-guided applications. **(D)** [^177^Lu]Lu-3BP-227, shown as a representative therapeutic antagonist-based radioligand. Only selected representative structures are shown in the main text to preserve the translational focus of this review, whereas additional probe structures are provided in the **Supplementary Information**.

Neurotensin (NT) is the endogenous ligand of neurotensin receptor 1 (NTS1) (13). It is an endogenous linear peptide composed of 13 amino acids, with the primary sequence pGlu-Leu-Tyr-Glu-Asn-Lys-Pro-Arg-Arg-Pro-Tyr-Ile-Leu (14). Previous studies have shown that the C-terminal hexapeptide fragment, NT (8–13) [Arg8-Arg9-Pro10-Tyr11-Ile12-Leu13], retains the core structural features required for receptor recognition and for mediating the major biological effects. It is therefore widely regarded as the key pharmacophore of NT (15, 16). Representative structures of NT and its core pharmacophore NT (8–13) are shown in **Scheme 1A**. Based on this structural framework, most currently available NTS1-targeted peptide probes are derived from NT (8–13). Functional imaging and targeted therapeutic applications are achieved by introducing fluorescent moieties, radionuclide chelators, or other functional modules into the peptide backbone (17–19). However, native NT and its short peptide derivatives are rapidly degraded by peptidases *in vivo*, resulting in a short plasma half-life (20, 21). This characteristic markedly limits the effective exposure of the intact ligand at the tumor site and further affects tumor uptake, imaging contrast, and the delivery efficiency of therapeutic radionuclides (22). Therefore, preserving the receptor recognition capability of NT (8–13) while improving *in vivo* stability and optimizing pharmacokinetic behavior through structural modification has become a major focus in the development of NTS1-targeted probes (23, 24).

NTS1 is a high-affinity G protein-coupled receptor (GPCR) that is highly expressed across multiple human malignancies, including pancreatic ductal adenocarcinoma (PDAC) and cancers of the colorectum, prostate, breast, and lung, while its expression remains relatively low in normal tissues (25–29). In addition, NTS1-related signaling pathways are closely associated with malignant phenotypes such as tumor cell proliferation, migration, invasion, and angiogenesis, further supporting its potential as a target for cancer diagnosis and therapy (30, 31). With the tumor-targeting value of NTS1 increasingly recognized, elucidating its ligand recognition patterns and conformational regulatory mechanisms has become essential for guiding the rational design and performance optimization of targeted probes. In recent years, structural biology has elucidated the complex structures of NTS1 bound to ligands (e.g., neurotensin analogs) and signaling molecules (e.g., β-arrestin), revealing its molecular mechanisms of action and providing critical support for the rational design of targeted probes (16, 32, 33). Krumm et al. demonstrated that the orthosteric binding pocket of NTS1, which serves as the primary site for endogenous ligands and classical agonists, possesses well-defined spatial and chemical features and exhibits dynamic conformational coupling with conserved intracellular linker residues. This finding helps explain how different ligands stabilize specific receptor conformations and thereby influence binding behavior (32). Subsequent studies on agonist binding modes (16), β-arrestin-associated conformations and allosteric regulatory sites (33) have further deepened our understanding of NTS1 conformational selectivity and signaling regulation. Together, these findings provide a molecular basis for optimizing probe affinity, selectivity, stability, and receptor-targeting properties, and support the continued development of NTS1-targeted probes for fluorescent, radionuclide, and radioligand imaging, as well as radioligand therapy.

This review summarizes the latest advances in NTS1-targeted molecular probes for the diagnosis and treatment of diverse malignancies, with an emphasis on their applications in tumor molecular imaging and targeted therapy. It also delineates current challenges in clinical translation and outlines future directions, aiming to provide a reference for precision cancer diagnosis and treatment. Representative NTS1-targeted molecular probes, including their structural features, receptor affinity, and major imaging and therapeutic applications, are summarized in **Table 1**.

**Table 1:** Representative NTS1-targeted molecular probes: structural features, affinity, and major imaging/therapeutic applications.

Representative probe	Probe type	Structural feature	Label/moiety	Affinity (cell model)	*In Vivo* tumor uptake (%ID/g or SUV, model, time point)	Main imaging/therapy mode	Reference
FITC-Ava-Arg-MeArg-Pro-Tyr-Tle-Leu-OH	Fluorescent Probe	NTS analog	FITC	IC_50_ = 479 nM (HT-29 cells)	—	Fluorescence imaging	(34)
Compound **6**	Fluorescent Probe	NTS analog	Indolinium-type cyanine dyes	pKi = 8.86 (CHO-hNTS1R cells)	—	Fluorescence imaging	(35)
Compound **21** (UR-FE094)	Fluorescent Probe	Peptide agonist	sulfo-Cy5	K_i_ = 0.094 nM (HT-29 cells)	—	Fluorescence imaging	(36)
IRDye800-NT	NIR Fluorescence Probe	NTS analog	IRDye800	IC_50_ = 16.66 nM (HT-29 cells)	8.09 ± 0.38 × 10^8^ (30 min), 6.67 ± 0.43 × 10^8^ (60 min) (p/s/cm²/sr)/(μW/cm²) (AsPC-1 model, fluorescence imaging)	NIR fluorescence imaging	(37)
[^68^Ga]Ga-DOTA-NT-20.3	PET Probe (^68^Ga)	Peptide agonist	^68^Ga	—	5.28 ± 0.93%IA/g (1 h, AsPC-1 model, biodistribution)	PET imaging	(38)
[^68^Ga]Ga-bisNODAGA-**16**	PET Probe (^68^Ga)	Non-peptide antagonist	^68^Ga	K_i_ = 9.05 nM (HT-29 cells)	4.917 ± 0.776%ID/g (2 h, HT-29 model, biodistribution)	PET imaging	(39)
[^68^Ga]**8**	PET Probe (^68^Ga)	Peptide agonist	^68^Ga	K_i_ = 20 nM (HT-29 cells)	1.55 ± 0.35%ID/g (1 h, HT-29 model, biodistribution)	PET imaging	(17)
[^68^Ga]UR-LS130	PET Probe (^68^Ga)	Peptide agonist	^68^Ga	K_i_ = 1.2 nM (HT-29 cells)	8.4 ± 2.9%ID/g (45 min, HT-29 model, biodistribution)	PET imaging	(24)
[^68^Ga]Ga-JMV6659	PET Probe (^68^Ga)	NTS analog	^68^Ga	Kd = 6.29 ± 1.37 nM (HT-29 cells)	7.8 ± 0.54%ID/g (2 h, HT-29 model, biodistribution)	PET imaging	(40)
[^68^Ga]Ga-TRAP(NT4)_3_	PET Probe (^68^Ga)	Peptide agonist	^68^Ga	Ki = 0.12 ± 0.03 nM (HT-29 cells)	1.74 ± 0.21%ID/g (60 min), 1.44 ± 0.13%ID/g (90 min) (HT-29 model, biodistribution)	PET imaging	(41)
[^68^Ga]Ga-DOTA-NT-20.3-IPBA	PET Probe (^68^Ga)	Peptide agonist	^68^Ga	Kd = 33.86 ± 5.86 nM (HT-29 cells)	2.67 ± 0.35%ID/g (30 min), 2.37 ± 0.21%ID/g (60 min), 2.07 ± 0.21%ID/g (120 min) (HT-29 model, PET ROI) SUVmax = 3.73 ± 1.22,SUVmean = 2.41 ± 0.59(60 min,human lung adenocarcinoma)	PET imaging	(42)
[^68^Ga]Ga-DOTA-NT-20.3-Ibu	PET Probe (^68^Ga)	Peptide agonist	^68^Ga	Kd = 30.32 nM (HT-29 cells)	4.96 ± 1.27%ID/g (30 min), 4.50 ± 0.93%ID/g (60 min), 3.99 ± 1.11%ID/g (120 min) (HT-29 model, PET ROI) SUVmax = 3.83 ± 1.55,SUVmean = 2.41 ± 0.67(60 min,human lung adenocarcinoma)	PET imaging	(43)
[^64^Cu]Cu-DOTA-NT	PET Probe (^64^Cu)	Peptide agonist	^64^Cu	IC_50_ = 2.9 nM (HT-29 cells)	1.27 ± 0.24%ID/g (4 h, HT-29 model, biodistribution)	PET imaging	(44)
[^64^Cu]Cu-NT-CB-NOTA	PET Probe (^64^Cu)	Non-peptide antagonist	^64^Cu	—	76.9% retention at 48h compared with uptake 1h (H1299 model)	PET imaging	(45)
[^64^Cu]Cu-4b	PET Probe (^64^Cu)	Non-peptide antagonist	^64^Cu	IC_50_ = 8.17 nM (H1299 cells)	9.57 ± 1.35%ID/g (4 h), 9.44 ± 2.38%ID/g (24 h), 9.72 ± 4.89%ID/g (48 h) (H1299 model, PET ROI)	PET imaging	(46)
[¹^8^F]FGlc-NT4	PET Probe (¹^8^F)	Peptide agonist	¹^8^F	K_i_ = 16 ± 2.8 nM (HT-29 cells)	2.0%ID/g (30 min), 1.2%ID/g (65 min) (HT-29 model, biodistribution)	PET imaging	(47)
[¹^8^F]**8**	PET Probe (¹^8^F)	Non-peptide antagonist	¹^8^F	K_i_ = 0.98 nM (CHO-hNTS1R)	0.84 ± 0.11%ID/g (10 min), 0.74 ± 0.14%ID/g (60 min) (HT-29 model, biodistribution)	PET imaging	(48)
[^55^Co]Co-NT-CB-NOTA	PET Probe (^55^Co)	Non-peptide antagonist	^55^Co	—	60.2% retention at 24 h compared with uptake 1 h (HT29 model)	PET imaging	(45)
[^55^Co]Co-NOTA-NT-20.3	PET Probe (^55^Co)	Peptide agonist	^55^Co	—	SUV 0.257 ± 0.055 (1 h), 0.300 ± 0.035 (4 h), 0.202 ± 0.012 (24 h) (HT-29 model, PET ROI)	PET imaging	(49)
[^55^Co]Co-NT-Sarcage	PET Probe (^55^Co)	Non-peptide antagonist	^55^Co	—	~5-7%ID/g (1–9 h), ~4-5%ID/g (24 h) (HT-29 model, PET ROI)	PET imaging	(50)
[^99m^Tc]Tc-NT-XI	SPECT Probe (^99m^Tc)	Peptide agonist	^99m^Tc	Kd= 0.5 nM (HT-29 cells)	9.1 × 10⁻^4^ %ID/g (18–22 h, pancreatic tumor, patient 4, ex vivo)	SPECT imaging	(51)
[^99m^Tc]Tc-Demotensin VI	SPECT Probe (^99m^Tc)	Peptide agonist	^99m^Tc	IC_50_ = 0.08 nM (WiDr cells)	Preclinical (WiDr model): 4.30 ± 0.45%ID/g (1 h), 2.31 ± 0.28%ID/g (4 h)	SPECT imaging	(52)
[^64^Cu]Cu-DOTA-NT-Cy5.5	Bimodal Probe (PET/Fluorescence)	Peptide agonist	^64^Cu, Cy5.5	IC_50_ = 0.65 nM (HT-29 cells)	1.91 ± 0.22%ID/g (1 h), 1.79 ± 0.16%ID/g (4 h) (HT-29 model, PET ROI)	PET imaging and intraoperative fluorescence navigation	(53)
[^68^Ga]Ga-NODAGA-Lys(Cy5)-AEEAc-[Me-Arg8,Tle12]-NT (7–13)	Bimodal Probe (PET/Fluorescence)	Peptide agonist	^68^Ga, Cy5	K_i_ = 12 nM (AsPC-1 cells)	2.56 ± 0.97%ID/g (1 h, AsPC-1 model, biodistribution)	PET imaging and intraoperative fluorescence navigation	(19)
[¹¹¹In]In-JMV7490	SPECT Probe (¹¹¹In)	Peptide agonist	¹¹¹In	Kd = 77.47 ± 14.06 nM (HT-29 cells)	5.86 ± 0.86%ID/g (1 h), 3.65 ± 0.29%ID/g (4 h) (HT-29 model, biodistribution)	SPECT imaging	(54)
							
[^225^Ac]di-DOTA-α,ϵ-Lys-NT (6–13)	Therapeutic Probe (α-emitter)	Peptide agonist	^225^Ac	IC_50_ = 5.0 nM (HT-29 cells)	—	α-emitter therapy	(55)
[^177^Lu]Lu-3BP-227	Therapeutic Probe (β-emitter)	Non-peptide antagonist	^177^Lu	IC_50_ = 0.59 nM (HT-29 cells)	Preclinical (HT-29): 19.0 ± 3.6%ID/g (3 h), 8.6 ± 4.0%ID/g (20 h), 2.7 ± 1.6%ID/g (69 h)	β-emitter therapy	(56, 57)

Affinity values and *in vivo* uptake data were reported as in the original studies and may not be directly comparable because of differences in assay format, cell model, imaging modality, and time point. Abbreviations: PET, positron emission tomography; SPECT, single-photon emission computed tomography; NIR, near-infrared. Representative chemical structures of selected NTS1-targeted probes are shown in **Scheme 1** and **Supplementary Schemes S1**-**S2**.

Bold values indicate numerals formatted in accordance with the radiopharmaceutical nomenclature guidelines.

## 2 Applications of NTS1-targeted molecular probes in tumor molecular imaging

Molecular imaging enables dynamic monitoring, functional characterization, and precise quantification of specific biological targets and pathophysiological processes at the cellular and molecular levels, thereby providing a noninvasive approach for early diagnosis, staging, treatment response assessment, and prognostication in oncology (58–60). The success of molecular imaging depends on selecting targets with high tumor-to-normal tissue contrast, accessible presentation, and biological stability. NTS1 is overexpressed in numerous tumors, yet exhibits low expression in most normal tissues (25–29), making it an attractive diagnostic target. Moreover, ligand binding to NTS1 and subsequent receptor-mediated endocytosis and cellular retention amplify signal accumulation, highlighting applications ranging from intraoperative optical probes for surgical margin delineation to PET/SPECT tracers for systemic staging and therapeutic monitoring (61–64).

### 2.1 Optical imaging

Optical imaging, particularly fluorescence imaging, holds a significant position in tumor molecular imaging due to its advantages of high sensitivity, real-time capability, non-ionizing radiation, and relative affordability (65, 66). NTS1-targeted fluorescent probes are primarily used to visualize receptor expression at the cellular and tissue levels and to enable fluorescence-guided surgery. Early designs typically conjugated fluorophores to NT peptides or their derivatives. Maes et al. introduced a thiourea moiety at the N-terminus of an NT (8–13) analog and labeled it with fluorescein isothiocyanate (FITC). The resulting probe exhibited receptor-mediated endocytosis in HT-29 cells (34), suggesting that fluorescently labeled NT derivatives could be used for intraoperative tumor margin delineation. However, these early probes generally show reduced receptor affinity, especially in analogues with improved metabolic stability. This limitation compromises their *in vivo* performance, as decreased affinity reduces tumor-targeting efficiency. In addition, the short emission wavelength of fluorescent moieties further limits imaging performance in deep tissues.

**Figure 1:**
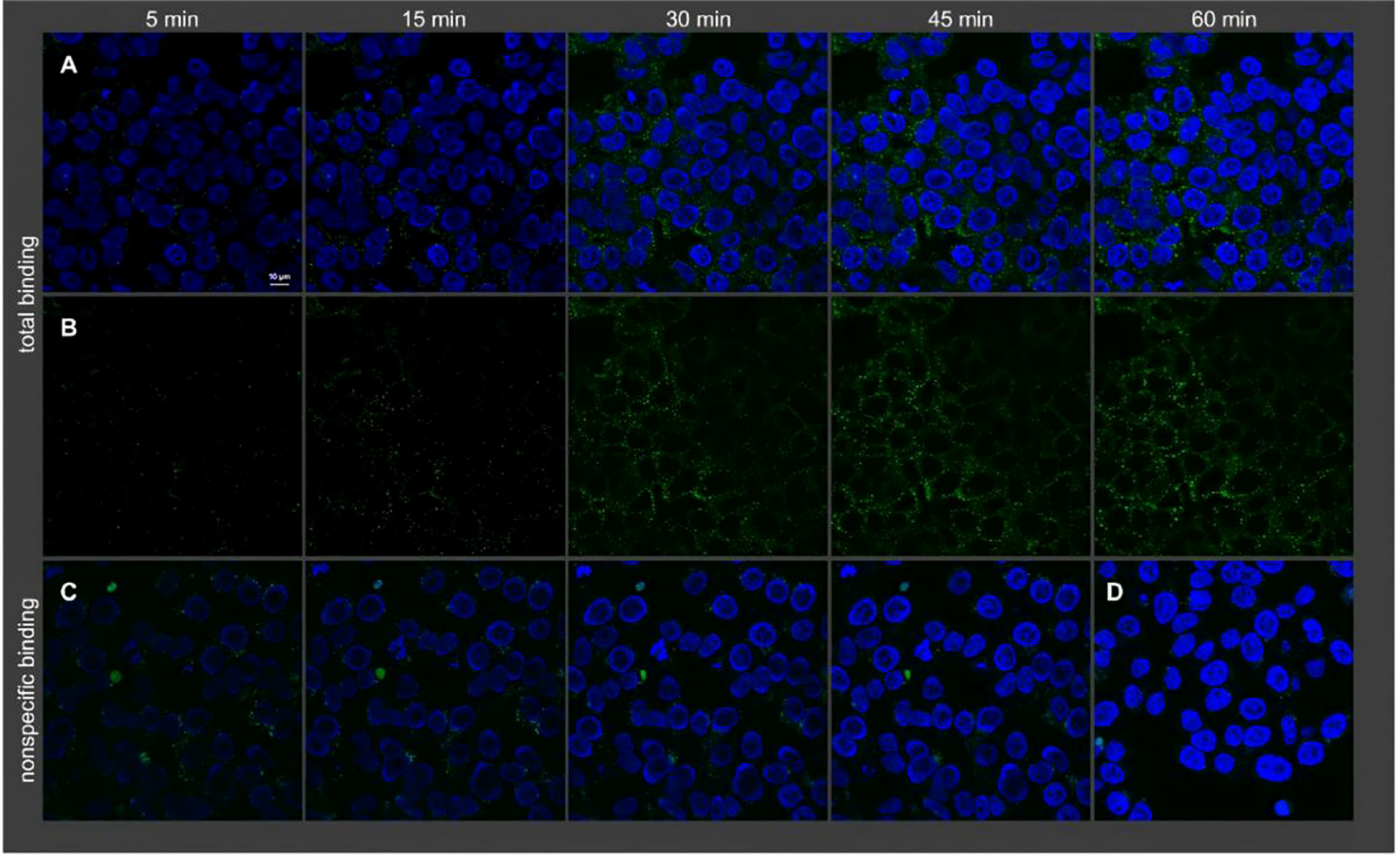
Visualization of binding of 19 (2 nM) to intact HT-29 cells (temperature: 22 °C) by confocal microscopy. Shown is total binding **(A, B)**, nonspecific binding **(C)**, and autofluorescence **(D)**. Nuclei were stained with H33342 (2 μg/mL). **(A)** Merged fluorescence of 19 (green) and nuclei (blue). **(B)** Fluorescence of 19, without nuclei. **(C)** Merged fluorescence of 19 and nuclei acquired in the presence of 1 μM NT(8−13). Copyright ^©^ 2025 The Fabian J Ertl, Anna Friedel, Elena J Schmid, Carina Höring, Nataliya Archipowa, Pierre Koch, Simone Maschauer, Roger J Kutta, Olaf Prante, Max Keller. Published by American Chemical Society.

**Figure 2:**
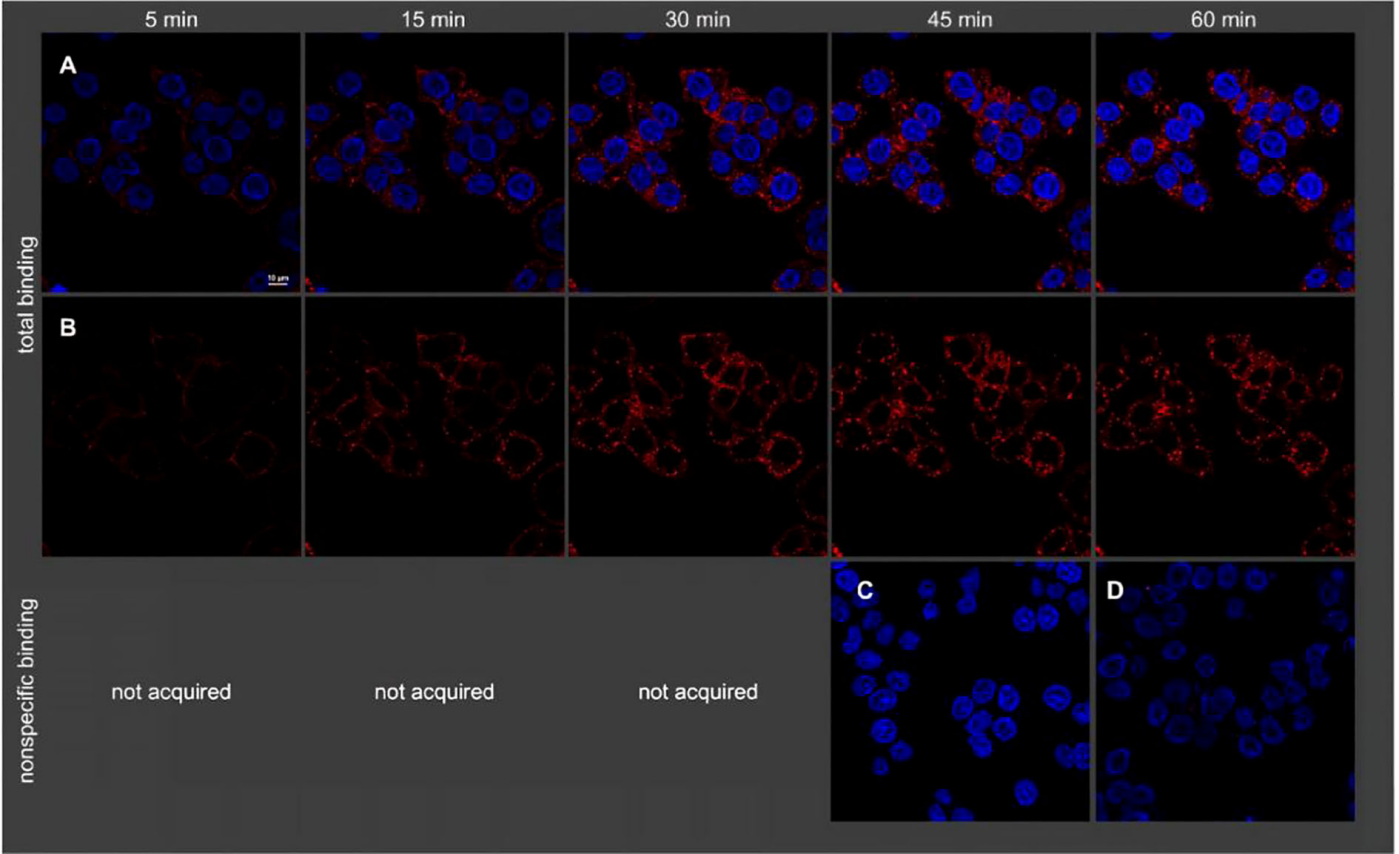
Visualization of binding of 21 (2 nM) to intact HT-29 cells (temperature: 22 °C) by confocal microscopy. Shown is total binding **(A, B)**, nonspecific binding **(C)**, and autofluorescence **(D)**. Nuclei were stained with H33342 (2 μg/mL). **(A)** Merged fluorescence of 21 (red) and nuclei (blue). **(B)** Fluorescence of 21, without nuclei. **(C)** Merged fluorescence of 21 and nuclei acquired in the presence of 1 μM NT(8−13). Note: as no nonspecific binding of 21 was observed, images of nonspecific binding were only acquired after 45 min. Copyright ^©^ 2025 The Fabian J Ertl, Anna Friedel, Elena J Schmid, Carina Höring, Nataliya Archipowa, Pierre Koch, Simone Maschauer, Roger J Kutta, Olaf Prante, Max Keller. Published by American Chemical Society.

With advances in probe design and synthetic methodologies, researchers have sought to develop NTS1-targeted fluorescent probes that combine higher affinity and stability with superior optical performance. Keller et al. synthesized a series of high-affinity NTS1 probes by attaching red fluorophores (indolenine- or pyridinium-based cyanines) to the arginine residue. Compared with conventional N-terminally labeled NT analogs, probes generated by this strategy showed improved performance, including higher binding affinity (pKi = 8.15–9.12 in intact CHO-hNTS1R cells), full agonist activity, good stability, and low background signal (35). These probes are powerful tools for *in vitro* studies of NTS1 at the cellular and tissue levels; their high affinity ensures effective receptor recognition and binding. Recently, Ertl et al. developed peptide-based fluorescent NTS1 ligands using a triple-modification strategy that exhibit excellent stability (t_1/2_ > 48 h) and very high affinity (Ki = 0.094 nM in intact HT-29 cells). The representative chemical structure of Compound 21 (UR-FE094) is shown in **Supplementary Scheme S2**. These ligands are suitable for competitive binding assays and fluorescence imaging to visualize NTS1 expression in cells and tumor tissues, as illustrated in **Figures 1**, **2** (36). Co-optimizing affinity and stability is pivotal in probe design, as these parameters directly determine *in vivo* efficacy and persistence. High affinity enables effective receptor binding even at low receptor expression levels, whereas high stability reduces *in vivo* degradation and prolongs tumor-site residence time, thereby improving imaging quality.

In addition to directly labeled fluorescent probes, researchers have pursued alternative strategies to enhance optical imaging of NTS1. Building on SBI-553, an established intracellular allosteric modulator of NTS1, Vogt et al. designed and synthesized a novel fluorescent probe (probe 14) that binds the intracellular allosteric site of NTS1 with high affinity. Using this probe, they established a NanoBRET-based nonradioactive ligand-binding assay. This assay not only facilitates efficient discovery and optimization of modulators targeting the NTS1 allosteric pocket but also exploits the probe’s allosteric mechanism to indirectly assess interactions between the endogenous orthosteric ligand and the receptor. Through probe-enabled library screening, the team identified multiple novel chemotypes with affinities in the micromolar range, providing experimental support for NTS1-targeted lead discovery in pain and addiction disorders (67). This strategy extends the target space to intracellular allosteric sites and provides a new tool for studying receptor dynamics and endogenous ligand interactions. However, achieving sufficient probe delivery to NTS1-expressing cell membranes *in vivo* remains challenging, which substantially limits its practical utility as an imaging agent and currently restricts its application to cell-based or ex vivo mechanistic studies.

**Figure 3:**
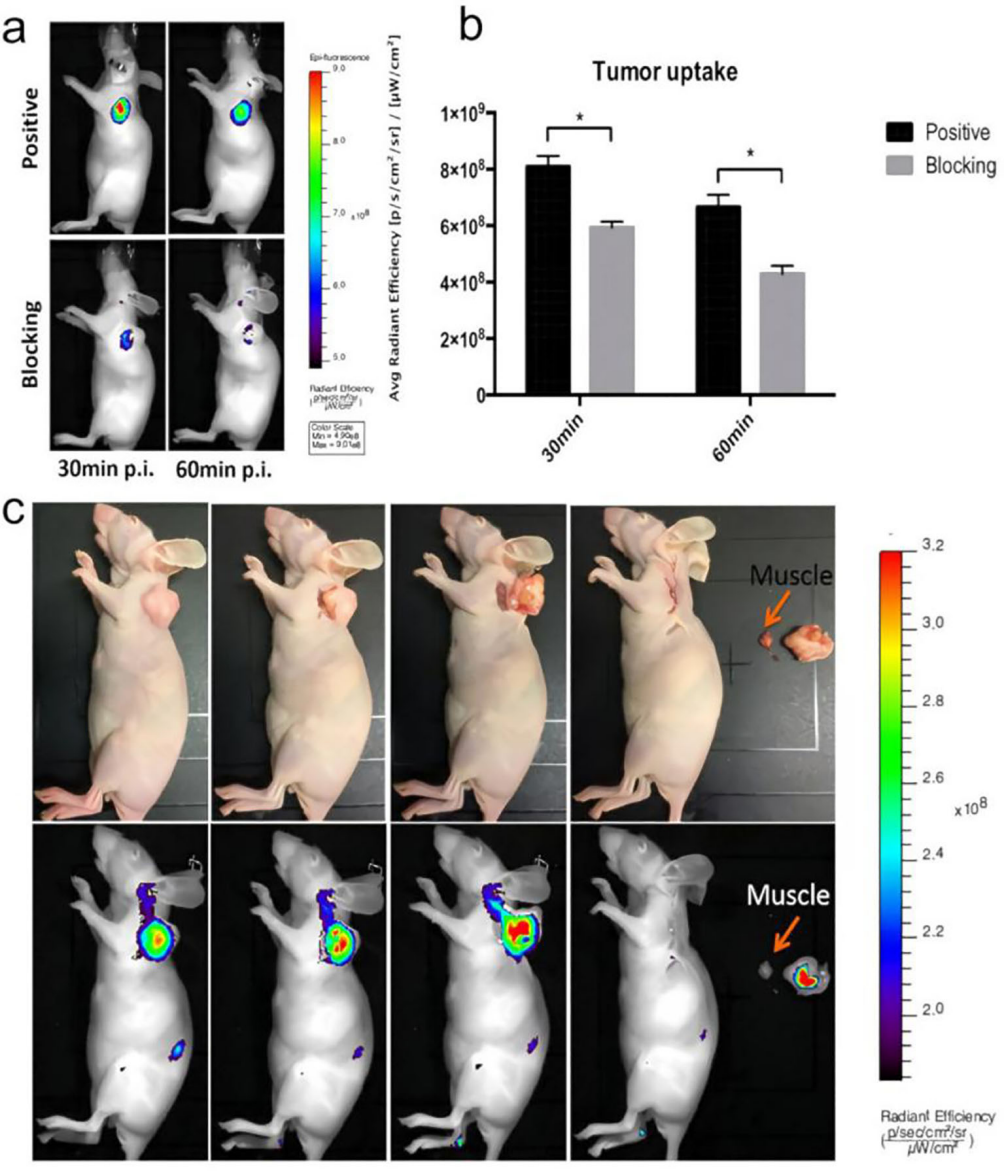
**(a)** Representative *in vivo* fluorescent images of AsPC1 tumor-bearing mice at 30 min and 60 min post injection of NT-IRDye800 showed clear tumor contrast in the positive group, and the tracer specificity was confirmed by the blocking group (n=3/group). **(b)** The quantitative tumor uptakes derived from fluorescent imaging were significantly (P<0.05) different at both time points in the positive and blocking groups. **(c)** Mouse bearing an AsPC1 tumor at 1 h post-injection of NT-IRDye800. The tumor could be successfully removed using fluorescence-guided surgery. The upper row shows digital images from the surgery, and the lower row shows fluorescent images. Copyright ^©^ 2017, Springer-Verlag Wien.

Near-infrared (NIR) fluorescence imaging, particularly in the second NIR window (NIR-II, 1000–1700 nm), has become a major focus in optical imaging because of its deeper tissue penetration, reduced background autofluorescence, and higher signal-to-noise ratio (68–71). Deng et al. developed a dual-modality PET/fluorescence probe, [^64^Cu]Cu-DOTA-NT-Cy5.5, for molecular imaging of NTS1-positive tumors. In an HT-29 xenograft model, the probe exhibited pronounced tumor uptake and produced high-contrast fluorescence signals, indicating its potential to enable fluorescence-guided surgery in NTS1-positive patients (53). In a study of pancreatic ductal adenocarcinoma (PDAC), Yin et al. employed IRDye800-NT for near-infrared fluorescence imaging, achieving high tumor-to-background contrast and validating fluorescence-guided surgery (**Figure 3**) (37). These studies indicate that NTS1-targeted near-infrared fluorescent probes hold promise for intraoperative tumor margin delineation and resection guidance, potentially enabling more precise tumor removal and reducing the incidence of positive margins (72–74). However, limited tissue penetration remains a major challenge for deep-seated, large, or anatomically complex solid tumors. In addition, clinical translation is restricted by the high cost and limited availability of detection equipment, as well as by the persistent effects of light scattering and absorption in deep tissues. As a result, the generalizability and reliability of this surgical navigation strategy still need to be validated in larger, clinically relevant studies.

Although optical imaging offers many advantages, its limited penetration depth in deep tissues remains a major challenge. To overcome this limitation, researchers are exploring activation-gated molecular probes and developing NIR-II imaging technology. These probes emit fluorescence only upon encountering specific biomarkers or microenvironmental stimuli, thereby substantially improving the signal-to-noise ratio (SNR) and detection specificity (75–79). Although direct studies on activatable optical probes targeting NTS1 are still limited, the receptor’s selective expression in the tumor microenvironment makes the development of such probes a promising approach to enhance NTS1-targeted optical imaging.

### 2.2 Nuclear medicine imaging

Nuclear medicine imaging, a noninvasive molecular imaging modality, enables early cancer diagnosis, precise staging, evaluation of treatment response, and surveillance for recurrence by targeting receptors or proteins selectively expressed on the surfaces of tumor cells (80–83). From an imaging physics standpoint, radionuclide-based molecular imaging primarily comprises single-photon emission computed tomography (SPECT) and positron emission tomography (PET). Both modalities provide high sensitivity, enable robust quantification, and achieve superior deep-tissue penetration, making them essential tools for tumor molecular imaging (84, 85). In this context, NTS1-targeted radiopharmaceuticals show considerable promise. Diagnostic radionuclides commonly used for labeling NTS1-targeted probes include ^68^Ga, ^18^F, ^64^Cu, ^55^Co, ^99m^Tc, and ^111^In. These radionuclides differ in emission type, physical half-life, and imaging compatibility, which directly influence the imaging time window and the clinical application of the probes (86). Among them, ^68^Ga and ^18^F are widely used positron-emitting PET isotopes, with half-lives of approximately 67.7 min and 109.8 min, respectively. ^68^Ga can be conveniently produced from a generator and is suitable for peptide probes with rapid clearance (87), whereas ^18^F provides higher imaging resolution and is better suited for centralized production and cross-center distribution (88). ^64^Cu and ^55^Co can also be used for PET imaging. With half-lives of approximately 12.7 h and 17.5 h, respectively, they are more suitable for delayed imaging, long-term pharmacokinetic evaluation, and assessment of tumor retention (89, 90). In contrast, ^99m^Tc and ^111^In are classic SPECT isotopes. ^99m^Tc mainly emits gamma rays and has a half-life of approximately 6 h, supporting its broad clinical use (91). ^111^In has a half-life of about 2.8 d and is used not only for SPECT imaging but also for pre-therapeutic biodistribution and dosimetry assessment (92, 93). Overall, the selection of diagnostic radionuclides depends not only on the requirements of PET or SPECT imaging but also on their compatibility with ligand circulation time, metabolic stability, and tumor retention characteristics.

#### 2.2.1 ^68^Ga-labeled NTS1-targeted PET imaging probes

**Figure 4:**
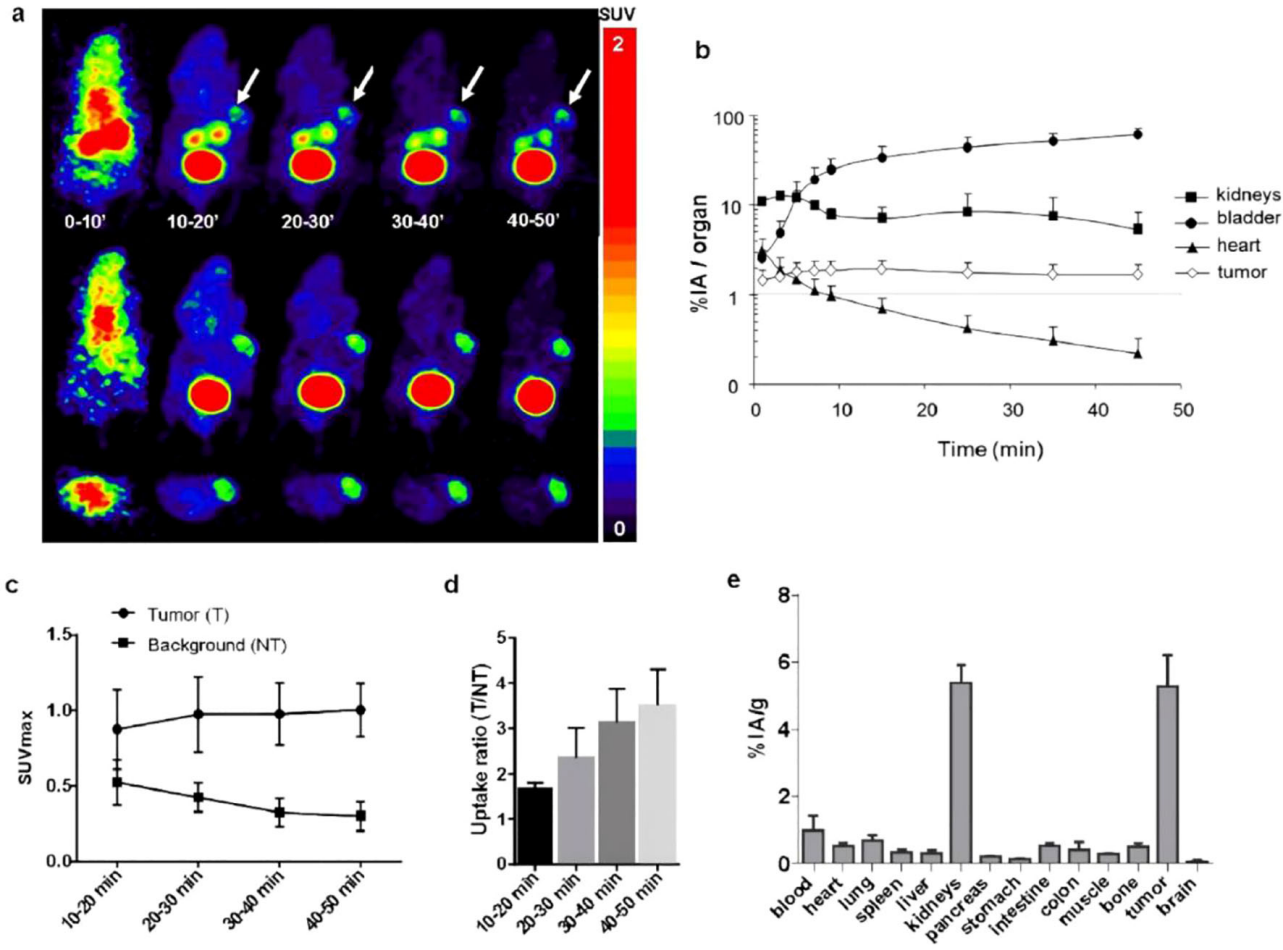
**(a)** Representative PET imaging of AsPC-1 tumor-bearing mice injected with 3 ± 0.8 MBq; (423 ± 53 pmol) of [^68^Ga]Ga-DOTA-NT-20.3 (n=4). Dynamic acquisition was started immediately p.i. over 50 min. PET imaging was composed of 5 frames of 1 min computed in the same image and 4 frames of 10 min. Upper image: maximum intensity projection (MIP), middle image: coronal slice, lower image: transversal slice. Tumor uptake of [^68^Ga]Ga-DOTA-NT-20.3 is clearly visible (arrow). **(b)** Time activity curve analysis of dynamic PET imaging, data expressed in mean % of injected dose in the volume of interest (VOI) (%ID) ± SD (n=4). The data were obtained by quantitative analysis of dynamic PET images of AsPC-1 tumor-bearing mice at 0–50 min p.i. of [^68^Ga]Ga-DOTA-NT-20.3 (n=4). **(c)** Time point of maximal standardized uptake value in tumor and background ROIs. Data expressed in mean SUVmax ± SD (n=4). **(d)** PET imaging analysis in tumor to non-tumor uptake ratio in mean ± SD (n=4). **(e)** ex vivo biodistribution of [^68^Ga]Ga-DOTA-NT-20.3 in AsPC-1 tumor-bearing nude mice at 1 h p.i. Tissue radioactivity is expressed as the percentage of injected activity per gram (%IA/g, mean ± SD). Copyright ^©^ 2019, American Chemical Society.

^68^Ga is a commonly used PET radionuclide and is well-suited for labeling peptide probes with short circulation times. For example, Marenco et al. first demonstrated *in vitro* that the novel probe [^68^Ga]Ga-DOTA-NT-20.3 exhibits substantial cellular uptake in the pancreatic ductal adenocarcinoma (PDAC) cell line AsPC-1 (94). The representative chemical structure of [^68^Ga]Ga-DOTA-NT-20.3 is shown in **Scheme 1B**. However, this study was limited to *in vitro* data, lacked *in vivo* evaluation, and did not include validation in a chronic pancreatitis model, leaving its *in vivo* imaging performance uncertain. Subsequently, Prignon et al. conducted preclinical evaluations showing that [^68^Ga]Ga-DOTA-NT-20.3 displays rapid tumor uptake, high contrast, and NTS1 specificity in animal models. Tissue microarray analysis indicated high NTS1 expression in PDAC (approximately 78%); biodistribution studies showed low uptake in normal pancreas but high uptake in tumors (5.28 ± 0.93%IA/g; **Figure 4**), and blocking experiments further confirmed receptor specificity. Importantly, [^68^Ga]Ga-DOTA-NT-20.3 differentiated PDAC from pancreatitis, indicating promise for PDAC molecular imaging (38) and practical relevance for clinical diagnosis. Collectively, these studies not only validate NTS1 as a feasible imaging target but also motivate further optimization of probe structure and *in vivo* performance.

Renard et al. synthesized a series of NTS1 antagonists bearing different chelators and labeled them with ^68^Ga for PET imaging. The results showed that the choice of chelator significantly affected the *in vivo* biodistribution of the probes, influencing tumor uptake, organ retention, and imaging contrast. Among these, [^68^Ga]Ga-bisNODAGA-16 exhibited favorable biodistribution, high tumor-to-organ ratios, and high-contrast PET images in HT-29 and AsPC-1 xenograft models, underscoring the critical role of the chelator in optimizing radiopharmaceutical performance (39). This finding indicates that effective probe design depends not only on the targeting ligand, but also critically on how the chelator’s chemical structure and conjugation mode determine key pharmacokinetic characteristics. However, such novel chelators may increase synthetic complexity and cost, and their compatibility with different metal radionuclides has not yet been systematically assessed. In addition, modifications to the chelator may alter probe hydrophilicity, thereby altering renal clearance pathways and remaining a major bottleneck for most peptide-based probes.

To improve NTS1-targeted probe performance and pharmacokinetics, various chemical modifications have been developed and systematically tested. For example, Maschauer et al. used solid-phase peptide synthesis (SPPS) to construct a series of NT peptide derivatives incorporating different chelators for ^68^Ga radiolabeling. *In vitro* competitive binding assays showed that these peptides displayed NTS1 affinity, with Ki values ranging from 19 to 110 nM in intact HT-29 cells. Biodistribution studies demonstrated that [^68^Ga]6 and [^68^Ga]8 achieved high tumor uptake in HT-29 tumor-bearing mice, and small-animal PET further confirmed their receptor specificity. Among them, [^68^Ga]8 showed a superior tumor-to-background ratio, making it particularly suitable for NTS1 PET imaging (17). These results indicate that optimization of the NT peptide structure can markedly influence receptor affinity and *in vivo* imaging performance, although high renal uptake remains a major barrier to clinical translation. Building on these findings, Schindler et al. optimized the molecular structure with a β,β-diMe-Tyr11 modification to construct a novel ^68^Ga-labeled neuropeptide vasopressin analog UR-LS130 ([^68^Ga]56). This approach substantially improved probe stability and tumor targeting, yielding high-contrast PET images in the HT-29 colorectal cancer mouse model (24). Iterative advances in molecular design have shifted the field’s focus from ligand screening to systematic pharmacokinetic optimization. High *in vivo* stability is crucial in radiopharmaceutical development because it limits degradation and clearance before the agent reaches the tumor, enhancing specific uptake and image quality.

Beyond site-specific amino acid modifications, Maschauer et al. were among the first to explore multimerization strategies by constructing a trimeric neurotensin analog, [^68^Ga]Ga-TRAP(NT4)_3_, using the TRAP chelator (41). This probe showed extremely high NTS1 affinity *in vitro* (0.12 ± 0.03 nM in intact HT-29 cells) and rapid internalization (> 90%), indicating that multimerization can markedly enhance receptor binding. However, these *in vitro* advantages did not fully translate into optimal *in vivo* imaging performance. In HT-29 colorectal tumor-bearing mice, the trimer achieved high tumor uptake (1.74%ID/g) but also showed substantial renal and hepatic retention (94.55%ID/g and 11.18%ID/g, respectively), which limited tumor-to-nontarget organ contrast. Thus, these findings suggest that although multimerization can improve receptor binding, it may also lead to unfavorable pharmacokinetic effects due to changes in molecular size, conformation, and clearance pathways, thereby restricting further translation. Building on this work, Fanelli et al. subsequently applied a silicon amino acid strategy by replacing natural amino acids with trimethylsilylalanine (TMSAla) to develop the 68Ga-labeled neurotensin analog [^68^Ga]Ga-JMV6659 (40). Compared with the TRAP multimer, [^68^Ga]Ga-JMV6659 showed lower *in vitro* affinity (Kd = 6.29 ± 1.37 nM in intact HT-29 cells) but demonstrated a more balanced profile in terms of plasma stability, receptor selectivity, and overall *in vivo* behavior. For example, in the HT-29 xenograft model, tumor uptake reached 7.8 ± 0.54%ID/g at 2 h post-injection and decreased to 1.38 ± 0.71%ID/g after blocking, indicating NTS1-mediated, specific tumor uptake. Taken together, TRAP multimerization and silicon amino acid substitution represent two distinct optimization strategies: the former emphasizes the affinity gain achieved through multimerization, whereas the latter focuses on a more balanced combination of affinity, stability, and *in vivo* distribution. This comparison indicates that maximizing *in vitro* affinity alone does not necessarily lead to superior *in vivo* imaging performance. Therefore, for NTS1-targeted PET probes, the balance among affinity, metabolic stability, and overall pharmacokinetics is often more important for clinical translation than the extreme improvement of any single parameter.

More recently, albumin−binding strategies have been adopted to prolong probe circulation time. Zhu et al. covalently linked the albumin−binding ligand IPBA (4−(p−iodophenyl)butyric acid) to the NTS1−targeting peptide to generate [^68^Ga]Ga−DOTA−NT−20.3−IPBA, thereby extending blood half−life and enhancing tumor accumulation and retention (42). Preclinical studies confirmed improved tumor uptake and extended retention, and early−phase clinical trials demonstrated favorable safety and clear tumor visualization in lung adenocarcinoma patients, highlighting its translational potential. The main advantage of this probe lies in the albumin “carrier effect,” which helps overcome the rapid clearance of small peptide probes. However, it should be noted that although this approach extends circulation time, it may also increase radiation exposure to normal tissues. Although no acute toxicity was observed in early studies, the long-term cumulative radiation dose still warrants careful monitoring.

Similarly, Liu et al. used ibuprofen (Ibu) as the albumin−binding moiety to produce [^68^Ga]Ga−DOTA−NT−20.3−Ibu (43). The representative chemical structure of this albumin-binding modified probe is shown in **Supplementary Scheme S1**. Preclinical results demonstrated enhanced tumor targeting, and initial clinical evaluations supported its suitability for imaging NTS1-expressing tumors. Collectively, these albumin−modified probes offer robust tools for clinical implementation of NTS1−targeted imaging. Overall, although current chemical modification and albumin-binding strategies have improved the performance of NTS1-targeted probes from different perspectives, the key determinants of *in vivo* pharmacokinetics remain incompletely understood. Clinical translation still faces common challenges, including high renal uptake, the need to balance metabolic stability with toxicity, and the management of inter-individual variability. Future studies should systematically clarify the relationship between modification strategies and pharmacokinetic behavior to provide more reliable molecular tools for precision diagnosis and therapy.

#### 2.2.2 ^64^Cu-labeled NTS1-targeted PET imaging probes

^64^Cu is a commonly used PET radionuclide with a relatively long half-life, making it well-suited for delayed imaging and long-term *in vivo* biodistribution studies. Deng et al. developed a dual-modality PET/fluorescence probe, [^64^Cu]Cu-DOTA-NT-Cy5.5, and systematically evaluated its PET performance. In HT-29 tumor models, the probe showed favorable *in vivo* behavior: at 1 and 4 h post-injection, it achieved high, specific tumor uptake with good retention, while tumor-to-background (e.g., muscle) contrast increased substantially, yielding clearer targeted images. Blocking studies verified that uptake was NTS1-mediated rather than nonspecific, supporting its utility for the noninvasive visualization and diagnosis of NTS1-positive tumors (53).

To further optimize probe performance, Deng et al. compared NT analogs conjugated to three ^64^Cu chelators—DOTA, NOTA, and AmBaSar. *In vitro* binding assays demonstrated that all three conjugates retained affinity for NTS1 at the cellular level. *In vivo* studies in HT-29 tumor models demonstrated that the NOTA- and AmBaSar-conjugated probes yielded higher tumor-to-background contrast than the DOTA-conjugated probe. PET imaging in PC3 xenograft models further confirmed their high tumor uptake, and the *in vivo* results aligned with the *in vitro* findings. Blocking studies further verified receptor-specific uptake (44). Overall, the study highlighted that chelator selection critically influences probe pharmacokinetics and image quality, suggesting that NOTA and AmBaSar may offer advantages over DOTA for optimizing ^64^Cu-labeled PET probes based on NT analogs. However, the *in vivo* metabolic stability of probes containing different chelators was not evaluated, nor was the effect of chelator charge differences on renal clearance analyzed. Recent studies have substantially improved the theranostic performance of NTS1 antagonists by incorporating the polyamine-containing macrocyclic chelator CB-NOTA and labeling the resulting construct with ^64^Cu (45). The rigid macrocyclic framework and polyamine functionalities of CB-NOTA allow modulation of the probe’s net charge and three-dimensional conformation, thereby enhancing interactions with the cell membrane. Consequently, tumor uptake is increased, intratumoral retention is prolonged (up to 48 h), and uptake in background organs is reduced. In NTS1-positive tumor models, the resulting molecular probe, [^64^Cu]Cu-NT-CB-NOTA, exhibited higher tumor-to-background contrast and more favorable pharmacokinetic profiles, underscoring the potential of polyamine macrocyclic chelators to precisely tune targeted probe performance and providing a new direction for optimizing NTS1-targeted theranostic probes.

**Figure 5:**
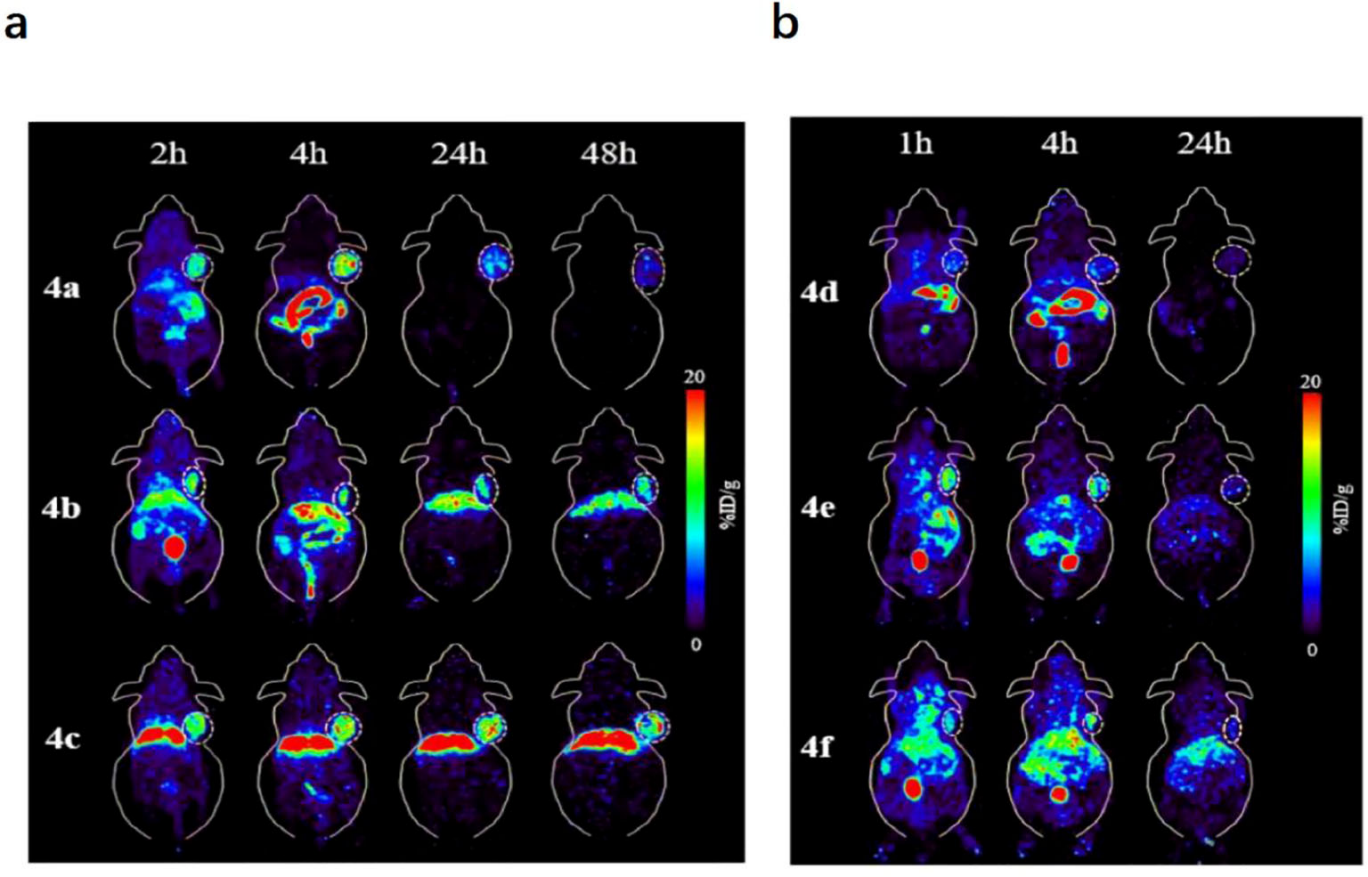
**(a)** Representative PET image of [^64^Cu]Cu-4a, [^64^Cu]Cu-4b, and [^64^Cu]Cu-4c in H1299 mouse at 2 h, 4 h, 24 h, and 48 h post-injection at 20%ID/g scale. **(b)** Representative PET image of [^64^Cu]Cu-4d, [^64^Cu]Cu-4e, and [^64^Cu]Cu-4f in H1299 mouse at 1h, 4h, and 24 h post-injection at 20%ID/g scale. Copyright ^©^ 2024, The Tao Zhang, Xinrui Ma, Muyun Xu, Jinghua Cai, Jianhua Cai, Yanguang Cao, Zhihao Zhang, Xin Ji, Jian He, German Oscar Fonseca Cabrera, Xuedan Wu, Weiling Zhao, Zhanhong Wu, Jin Xie, Zibo Li, under exclusive license to Springer-Verlag GmbH Germany, part of Springer Nature.

**Figure 6:**
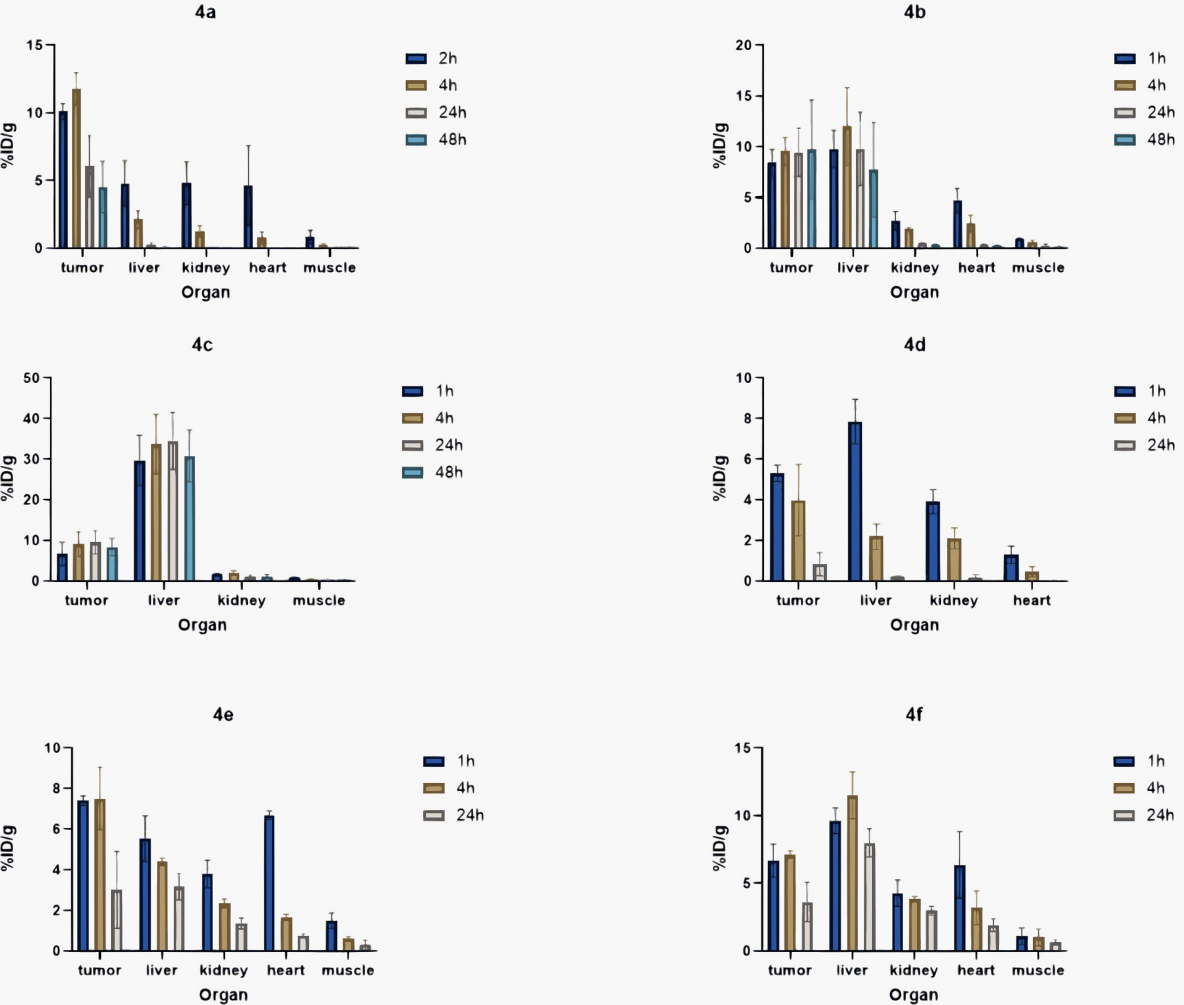
ROI uptake in major organs at 1 h/2 h, 4 h, 24 h, and 48 h post-injection after decay correction. Copyright ^©^ 2024, The Tao Zhang, Xinrui Ma, Muyun Xu, Jinghua Cai, Jianhua Cai, Yanguang Cao, Zhihao Zhang, Xin Ji, Jian He, German Oscar Fonseca Cabrera, Xuedan Wu, Weiling Zhao, Zhanhong Wu, Jin Xie, Zibo Li, under exclusive license to Springer-Verlag GmbH Germany, part of Springer Nature.

Building on chelator optimization, subsequent work extended to the systematic optimization of ligand architecture. Focusing on NTS1, Zhang et al. developed ^64^Cu-labeled agents with theranostic potential and systematically evaluated the effects of chelator identity and linker length on probe performance (46). They synthesized a series of NTS1 analogs incorporating propylamine (PA) linkers of varying lengths and different chelators, and evaluated them by PET/CT in an H1299 tumor model (**Figure 5**). Tumor uptake and tissue contrast depended strongly on the chelator; among the candidates, [^64^Cu]Cu-4b exhibited sustained, high tumor uptake with a manageable hepatic background (**Figure 6**). The authors also confirmed high NTS1 expression in patient tumor specimens and, based on combined imaging performance and therapeutic potential, designated [^64^Cu]Cu-4b as the lead candidate for further development. Collectively, these findings delineate the chelator’s critical role in determining *in vivo* distribution and image quality and, together with linker-length optimization, provide experimental evidence to guide the rational design of highly specific, high-contrast NTS1-targeted ^64^Cu PET probes. However, the experiments were conducted only in H1299 tumor-bearing mice and have not yet been validated in target tumors such as PDAC. Furthermore, the study indicated that excessively short linkers may impair tumor penetration, whereas overly long linkers may increase renal retention. Therefore, the optimal linker length remains to be investigated in the context of the tumor microenvironment.

#### 2.2.3 Other radionuclide-labeled NTS1-targeted PET/SPECT imaging probes

In addition to ^68^Ga and ^64^Cu, other radionuclides (e.g., ^18^F, ^55^Co, ^99m^Tc) have been applied to NTS1-targeted nuclear imaging. As a key PET radionuclide, ^18^F, with a physical half-life of approximately 109.8 min, provides a longer imaging window and facilitates intercenter distribution. Maschauer et al. used click chemistry to conjugate the peptide to a fluoroglycosyl moiety, prepared ^18^F-fluoroglycosylated NT (8–13) glycopeptide analogs, and evaluated these constructs in biodistribution and *in vivo* PET imaging studies (47). This strategy improved the metabolic stability of peptide-based probes, partially mitigating their rapid *in vivo* degradation, albeit at the cost of increased renal uptake. Subsequently, Lang et al. designed a nonpeptidic scaffold and, using click chemistry combined with glycoconjugation, introduced an ^18^F-labeled glucose azide to yield the first nonpeptidic NTS1 molecular imaging probe, [^18^F]**8**. This probe exhibited high affinity and stability, low renal uptake, and strong tumor retention, making it suitable for imaging NTS1-positive tumors (48).

^55^Co is a relatively new PET radionuclide with a half-life of approximately 17.5 h, longer than that of ^64^Cu, thereby enabling a broader imaging window. In a comparative study, Houston et al. reported that [55Co]Co-NOTA-NT-20.3 exhibited the highest cellular uptake (18.70 ± 1.30%ID/mg). *In vivo* PET/CT imaging further showed that the PET-derived tumor-to-heart standardized uptake value (SUV) ratio of [^55^Co]Co-NOTA-NT-20.3 at 24 h reached 20.28 ± 3.04, which was significantly higher than that of [^64^Cu]Cu-NOTA-NT-20.3. The tumor-to-blood ratio also increased over time, indicating an advantage of [^55^Co]Co-NOTA-NT-20.3 for NTS1-targeted imaging (49). Additionally, Lin et al. demonstrated that [^55^Co]Co-NT-Sarcage (an SR142948 derivative functionalized with DSar) exhibited high tumor uptake and a low background signal in HT-29 xenograft-bearing mice. Compared with [^64^Cu]Cu-NT-Sarcage, [^55^Co]Co-NT-Sarcage achieved a higher tumor-to-liver ratio while maintaining a broadly similar biodistribution, supporting the feasibility of Sar as a chelator for cobalt-based radiopharmaceuticals (50).

^99m^Tc, a prototypical radionuclide for SPECT, has a half-life of approximately 6 h and is widely used clinically, forming a cornerstone of nuclear medicine imaging. To address the rapid degradation of native NT, several studies introduced amino acid substitutions and optimized chelators to enhance the *in vivo* stability and receptor affinity of ^99m^Tc-labeled probes, followed by preclinical validation in HT-29 colon cancer, pancreatic cancer, and other xenograft models. These probes consistently demonstrated favorable performance, laying the groundwork for subsequent clinical translation (95–97). Clinical studies from the Buchegger and Maina groups indicate that ^99m^Tc-labeled NTS1-targeted radiotracers have a favorable safety profile; however, tumor detection rates are influenced by intratumoral NTS1 expression levels (51, 52). Notably, [^99m^Tc]Tc-Demotensin VI showed specific uptake in three patients with brain metastases from non–small cell lung cancer (NSCLC), which may reflect passive diffusion following disruption of the blood–brain barrier, indicating potential utility for imaging brain metastatic lesions (52). These translational studies underscore the promise of NTS1-targeted molecular probes in oncologic imaging. Future efforts should focus on developing probes with improved metabolic stability and prioritizing studies in tumors with high NTS1 expression, such as Ewing sarcoma.

Overall, NTS1-targeted radiotracers show considerable promise for the diagnostic evaluation of multiple tumor types, particularly pancreatic and prostate cancers. Hodolic et al. reported that radiolabeled NT has potential applications in PET imaging and therapy for patients with pancreatic cancer and suggested that it may aid in differentiating malignant tumors from inflammatory lesions (98). These radiotracers not only delineate tumor localization and volume but also enable the assessment of NTS1 expression, thereby informing treatment selection and response evaluation. However, nuclear medicine imaging still faces challenges, including exposure to ionizing radiation, high capital costs, and limited accessibility. Future studies should further optimize probe affinity, specificity, metabolic stability, and pharmacokinetic properties, and conduct larger, more rigorously designed clinical trials to validate diagnostic performance and clinical utility.

#### 2.2.4 Systematic comparison of molecular modification strategies and their potential for clinical translation

As noted above, the short plasma half-life and rapid *in vivo* degradation of NT analogues represent major barriers to their clinical translation (49, 99). For NTS1-targeted probes, these limitations not only reduce the proportion of intact ligand that reaches the tumor site but may also compromise tumor uptake, shorten lesion retention, affect tumor-to-background contrast, and compromise the efficient delivery of therapeutic radionuclides (24, 54). To overcome these challenges, researchers have developed various structural optimization strategies to improve metabolic stability and overall pharmacokinetic performance while preserving receptor affinity and targeting specificity to the greatest extent possible. Although these strategies act through different mechanisms, their shared goal is to optimize *in vivo* distribution and clearance, thereby improving imaging quality and expanding clinical applicability. The major design strategies and their effects on *in vivo* performance and clinical applicability are comparatively summarized in **Table 2**.

**Table 2:** Comparative summary of design strategies and key *in vivo* performance features of NTS1-targeted tracers.

Design strategy	Representative tracer (s)	Pharmacokinetic/stability features	Tumor uptake (%ID/g or SUV, model, time point)	Key off-target organ uptake/contrast	Clinical applicability
Chelator optimization (64Cu platform)	[^64^Cu]Cu-AmBaSar-NT [^64^Cu]Cu-DOTA-NT [^64^Cu]Cu-NOTA-NT	Stability: PBS RCP >98.9% intact at 30 min and >96.8% for AmBaSar/DOTA, 93.7% for NOTA at 4 h. PK: LV blood pool (^64^Cu-NOTA-NT) 26.77% injected dose at 15 s and 1.91%ID/g at 20 min; predominantly renal clearance.	1.70 ± 0.44/1.27 ± 0.24/1.64 ± 0.14%ID/g (HT-29 xenografts, 4 h; AmBaSar/DOTA/NOTA, respectively)	Kidney: 6.71 ± 0.75/4.28 ± 0.92/6.51 ± 0.63%ID/g	Improved image contrast; organ-burden trade-off
Chelator optimization (68Ga platform)	[^68^Ga]Ga-bisNODAGA-**16**	Stability: NOTA/NODAGA/THP >99% intact at 4 h; DOTA/DOTAGA ~80% intact. PK: 2 h p.i.; blood 0.393 ± 0.108%ID/g; kidneys 3.377 ± 0.425%ID/g; predominantly renal clearance.	4.917 ± 0.776%ID/g (HT-29 xenografts, 2 h)	Kidney: 3.377 ± 0.425%ID/g; Ratio: T/B = 13.2 ± 4.2	Short-lived PET suitability; chelator-dependent performance
Radiolabeling optimization (68Ga platform)	[^68^Ga]**8**	Stability: ≥tab intact at 60 min; no degradation product for [^68^Ga]8. PK: Rapid blood-pool clearance; T/B = 31 at 60 min.	1.55 ± 0.35%ID/g (HT-29 xenografts, 60 min)	Kidney: 44.59 ± 6.46%ID/g; Ratio: T/B = 31; Blocking: tumor 0.21 ± 0.06%ID/g	Rapid specific imaging; renal-uptake limitation
Linker optimization	[^64^Cu]Cu-4a [^64^Cu]Cu-4b [^64^Cu]Cu-4c	Stability: - PK: increasing linker length markedly increased liver uptake.	6.06 ± 2.28/9.44 ± 2.38/9.51 ± 2.88%ID/g (H1299 xenografts, 24 h)	–	Biodistribution tuning; liver-uptake trade-off
Peptide glycosylation	[¹^8^F]FGlc-NT4	Stability: >90% intact in serum; >98% *in vivo*. PK: Fast blood clearance; t_1_/_2_; not reported.	2.0/1.2%ID/g (HT-29 xenografts, 30/65 min)	Ratio: T/B = 1.7 to 3.5	Lower hepatobiliary background; modest retention gain
Peptide sequence stabilization	[¹¹¹In]In-JMV7490	Stability: 76% and 61% intact at 2 and 4 h ex vivo. PK: high internalization (85% at 1 h; 76% at 4 h) with reduced efflux.	5.86 ± 0.86/3.65 ± 0.29%ID/g (HT-29 xenografts, 1/4 h)	Kidney: 65.29 ± 3.08/77.07 ± 7.97%ID/g	Improved stability and retention; preclinical stage
Non-peptidic scaffold with glycosylation	[¹^8^F]**8**	Stability: Blood radio-HPLC >98% intact at 10 min and 30 min. PK: Fast blood clearance; negligible renal uptake.	0.84 ± 0.11/0.74 ± 0.14%ID/g (HT-29 xenografts, 10/60 min)	Kidney: negligible; Ratio: T/B = 0.3 to 4.4	Metabolic stability with low renal burden; modest tumor uptake
Multimerization	[^68^Ga]Ga-TRAP(NT4)3	Stability: Blood HPLC at 5 min showed ~92% intact tracer. PK: Blood 0.13 ± 0.02%ID/g (60 min) and 0.04 ± 0.02%ID/g (90 min); extremely low blood pool.	1.74 ± 0.21/1.44 ± 0.13%ID/g (HT-29 xenografts, 60/90 min)	Kidney: 94.55 ± 10.84/102.37 ± 29.88%ID/g; Ratio: T/B = 13 to 36	Affinity enhancement; renal-retention limitation
Albumin-binding modification	[^68^Ga]Ga-DOTA-NT-20.3-Ibu	Stability: Stable up to 120 min (qualitative). PK: Blood intact 1.38 ± 0.16%ID/g at 120 min.	3.99 ± 1.11%ID/g (HT-29 xenografts, 120 min)	Kidney: 3.32 ± 0.94; Ratio: T/B = 3.32 ± 0.94	Prolonged circulation and exposure; dosimetry concern
Non-peptidic antagonist scaffold	[^177^Lu]Lu-3BP-227	–	19.0 ± 3.6/2.7 ± 1.6%ID/g (HT-29 xenografts, 3/69 h)	Kidney:T/K = 5.2 ± 2.5 to 15.9 ± 19.3 (3/69 h, ex vivo)	Theranostic potential; further validation needed
Radionuclide matching	[^55^Co]Co-NOTA-NT-20.3 [^64^Cu]Cu-NOTA-NT-20.3	Stability: [^64^Cu] >95% at 1/4/24 h and [^55^Co] >95% at 4/24 h. PK: [^55^Co] heart SUV 0.033 ± 0.002 (1 h), 0.015 ± 0.002 (4 h), and 0.004 ± 0.001 (24 h).	PET-derived tumor SUV: 0.257 ± 0.055/0.315 ± 0.081 (HT-29 xenografts, 1 h; [^55^Co]/[^64^Cu])	24 h T/H SUV ratio = 20.28 ± 3.04/6.52 ± 1.97 ([^55^Co]/[^64^Cu])	Kinetic-window matching; platform-dependent utility

RCP, radiochemical purity; p.i., post-injection; T/B, tumor-to-blood ratio; T/K, tumor-to-kidney ratio; T/H, tumor-to-heart ratio. Threshold-type values (e.g., >95% and >98%) and ratios without reported error terms are presented exactly as reported in the original publications and were not recalculated.

Bold values indicate numerals formatted in accordance with the radiopharmaceutical nomenclature guidelines.

Among these strategies, chelator selection, linker modulation, and glycosylation primarily regulate *in vivo* behavior by influencing coordination stability, net charge, hydrophilicity, and spatial conformation. For ^64^Cu-labeled probes, different chelators may preserve similar receptor affinity, but tumor-to-background contrast and hepatic and renal distribution vary, indicating that the chelator not only determines labeling feasibility but also directly shapes the probe’s *in vivo* pharmacokinetic properties (44). Building on this, introducing PA linkers of varying lengths allows finer balancing between tumor uptake, tissue penetration, and clearance from non-target organs (46), while glycosylation typically enhances hydrophilicity, improves *in vivo* distribution, and to some extent increases resistance to enzymatic degradation, reducing hepatic background for certain probes (47). Overall, these strategies induce relatively limited modifications to the parent structure, offer good process controllability, and are well-suited for fine-tuning diagnostic imaging probes. However, improvements in sustained tumor retention and long-term *in vivo* stability are generally modest.

Compared with the strategies described above, direct stabilization of the peptide sequence more effectively addresses the major limitations of natural neurotensin analogues, namely their short half-life and susceptibility to enzymatic cleavage, and therefore has a more direct influence on sustained tumor uptake. As noted above, β,β-diMe-Tyr modification and other non-natural amino acid substitutions have shown that moderate sequence optimization can improve plasma stability and tumor retention without significantly increasing molecular complexity (24, 40). More recently, Bodin et al. further demonstrated that [^111^In]In-JMV7490, developed using an N-terminal stabilization strategy, achieved tumor uptake values of 5.86 ± 0.86%ID/g and 3.65 ± 0.29%ID/g at 1 h and 4 h, respectively, in HT-29 xenograft models. These findings indicate that targeted protection of labile sites is a critical strategy for enhancing the translational potential of such probes (54).

In contrast, multimerization and albumin-binding strategies are mainly intended to further improve pharmacokinetic behavior. Multimerization can markedly enhance receptor affinity and cellular binding, but these *in vitro* advantages do not necessarily translate into optimal *in vivo* imaging performance. Trimeric neurotensin analogues, represented by TRAP-based multimers, exhibit extremely high NTS1 affinity and rapid internalization, yet are also associated with pronounced renal and hepatic retention, which limits tumor-to-nontarget organ contrast and hinders further translation (41). These findings indicate that although multimerization can enhance apparent affinity, it may also lead to unfavorable pharmacokinetic effects because of changes in molecular size, conformation, and clearance pathways. By contrast, albumin-binding strategies prolong circulation time and increase tumor exposure and retention, providing an alternative approach to overcoming the rapid clearance of small peptide probes. Whether IPBA or ibuprofen is used as the albumin-binding moiety, studies have shown that these modifications enhance tumor uptake and prolong the effective imaging window, with some probes demonstrating clinical feasibility in early human studies (42, 43). However, prolonged circulation, although beneficial for tumor accumulation, may also increase radiation exposure to normal tissues. Therefore, the true value of these strategies depends on whether they can provide sufficient imaging or therapeutic benefit relative to the burden on non-target organs. From a longer-term perspective, non-peptidic antagonist scaffolds may represent an alternative and potentially more promising optimization pathway. By eliminating the enzymatically labile peptide backbone, this strategy can improve metabolic stability and clearance behavior at the structural level. Studies of both [18F]**8** (48) and [^111^In]In-3BP-227 (100) suggest that high receptor affinity can be maintained while achieving a more favorable *in vivo* profile, thereby supporting subsequent diagnostic and therapeutic development.

Taken together, the available evidence indicates that the translational value of NTS1-targeted probes is determined less by receptor affinity alone than by the overall balance among metabolic stability, circulation time, clearance route, tumor retention, non-target organ uptake, and radionuclide matching. Chelator optimization, linker engineering, glycosylation, sequence stabilization, multimerization, albumin binding, and non-peptidic scaffold design can each improve selected aspects of probe performance, but each also introduces distinct trade-offs in biodistribution, background clearance, renal burden, and practical feasibility. Therefore, no single modification strategy is universally optimal for clinical translation. In general, optimization of chelators and physicochemical properties is better suited to improving tumor-to-background contrast and therefore better serves diagnostic imaging, whereas sequence stabilization and non-peptidic scaffold design may be more advantageous when sustained tumor retention and therapeutic applications are prioritized. Albumin-binding strategies are particularly useful when prolonged circulation and increased tumor exposure are desired, although the potential increase in radiation burden to normal tissues requires careful evaluation. Future development of NTS1-targeted probes should therefore move beyond single-parameter optimization and adopt a more integrated design framework that matches molecular structure, pharmacokinetic behavior, and radionuclide selection to the intended diagnostic or therapeutic purpose.

### 2.3 Multimodal molecular imaging

Multimodal molecular imaging combines two or more complementary imaging modalities to mitigate the limitations of any single technique and enable more comprehensive and accurate tumor characterization (101, 102). Significant progress has been made in developing NTS1-targeted multimodal probes, with efforts primarily focused on dual-modality PET/fluorescence imaging.

Fluorescence imaging (FI) offers high sensitivity and spatial resolution but is limited by shallow tissue penetration. By contrast, nuclear imaging offers near-unlimited tissue penetration, exceptional sensitivity, and robust quantification (103). Accordingly, PET/fluorescence dual-modality probes have become a major strategy in NTS1-targeted research. Their main advantage is the integration of preoperative whole-body staging with intraoperative real-time guidance, allowing accurate tumor localization before surgery while reducing the risk of missed lesions due to limited optical penetration, thereby providing a quantifiable basis for surgical decision-making (104–107). Renard et al. designed and evaluated a dual-modality imaging agent targeting NTS1, [^68^Ga]Ga-NODAGA-Lys(Cy5)-AEEAc-[Me-Arg8, Tle12]-NT (7–13), for PET and fluorescence imaging. The representative structure of this dual-modality probe, which combines PET imaging with fluorescence-guided capability, is shown in **Scheme 1C**. The probe showed high tumor uptake at 1 h post-injection and rapid clearance. Fluorescence imaging corroborated the PET findings, and fluorescence-guided resection was achieved in preclinical models (19). However, some limitations remain. Rapid clearance reduces the blood-pool background and shortens the waiting time for imaging, but it may also limit tumor signal retention, especially during prolonged surgical procedures or in poorly perfused tumors. Moreover, Deng et al. developed [^64^Cu]Cu-DOTA-NT-Cy5.5, which enabled PET- and fluorescence-guided surgery, further supporting the potential of NTS1-targeted, dual-modality probes for oncologic theranostics (53). Overall, PET/fluorescence dual-modality probes show clear promise for preoperative diagnosis and intraoperative navigation, although tumor retention and the clinical operating window still remain areas for further optimization.

**Figure 7:**
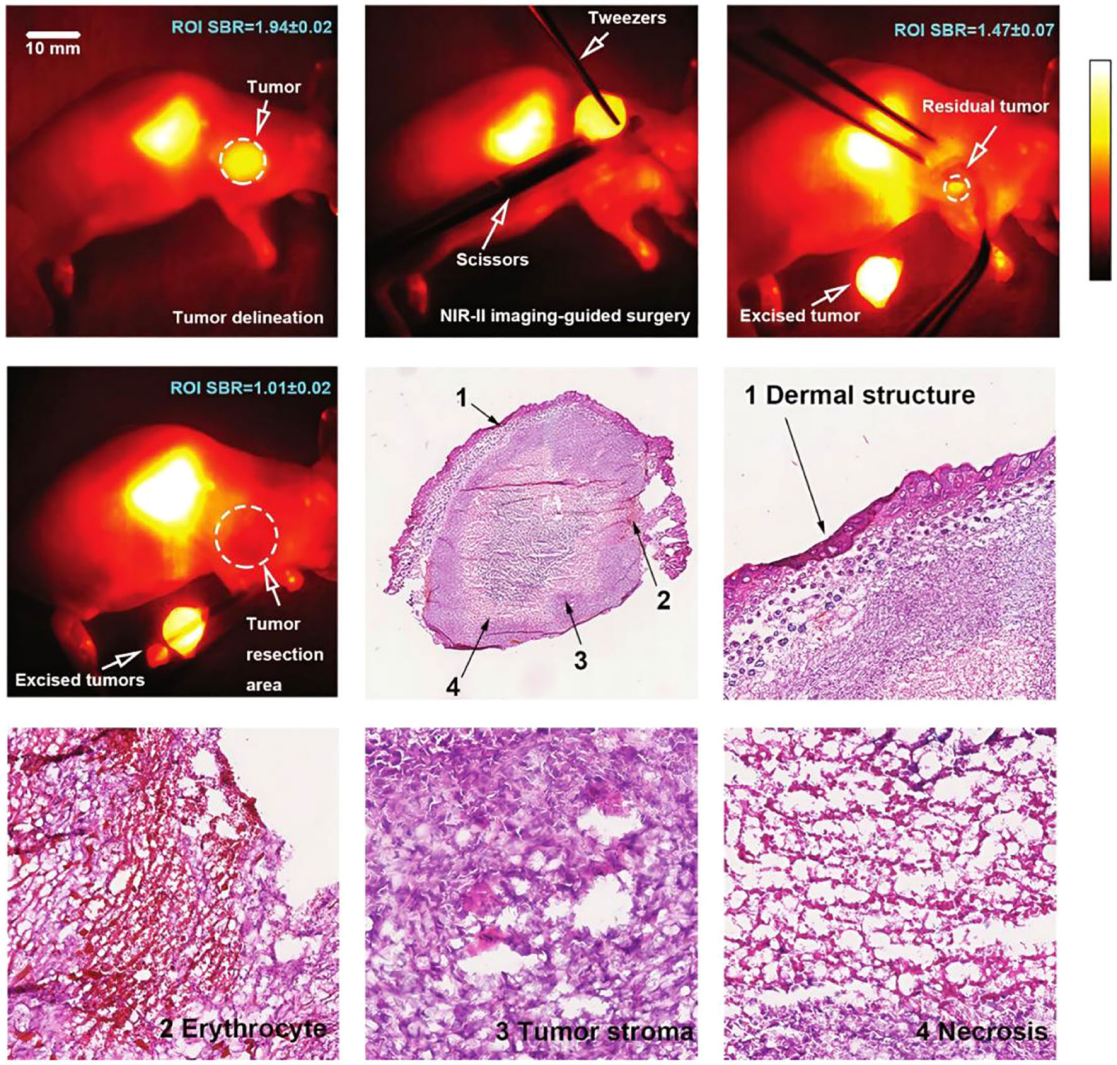
A431 tumor detection and NIR-II imaging (1000 nm longpass filter, exposure time 500 ms) guided surgery at 48 h post the vein injection of CH-4T/SLB–MSN–Mdot/Cu^2+^ nanoprobe. The colorbar ranges from 1134 to 21,949. The tumor was confirmed by H&E staining. ^©^ 2019 WILEY‐VCH Verlag GmbH & Co. KGaA, Weinheim.

**Figure 8:**
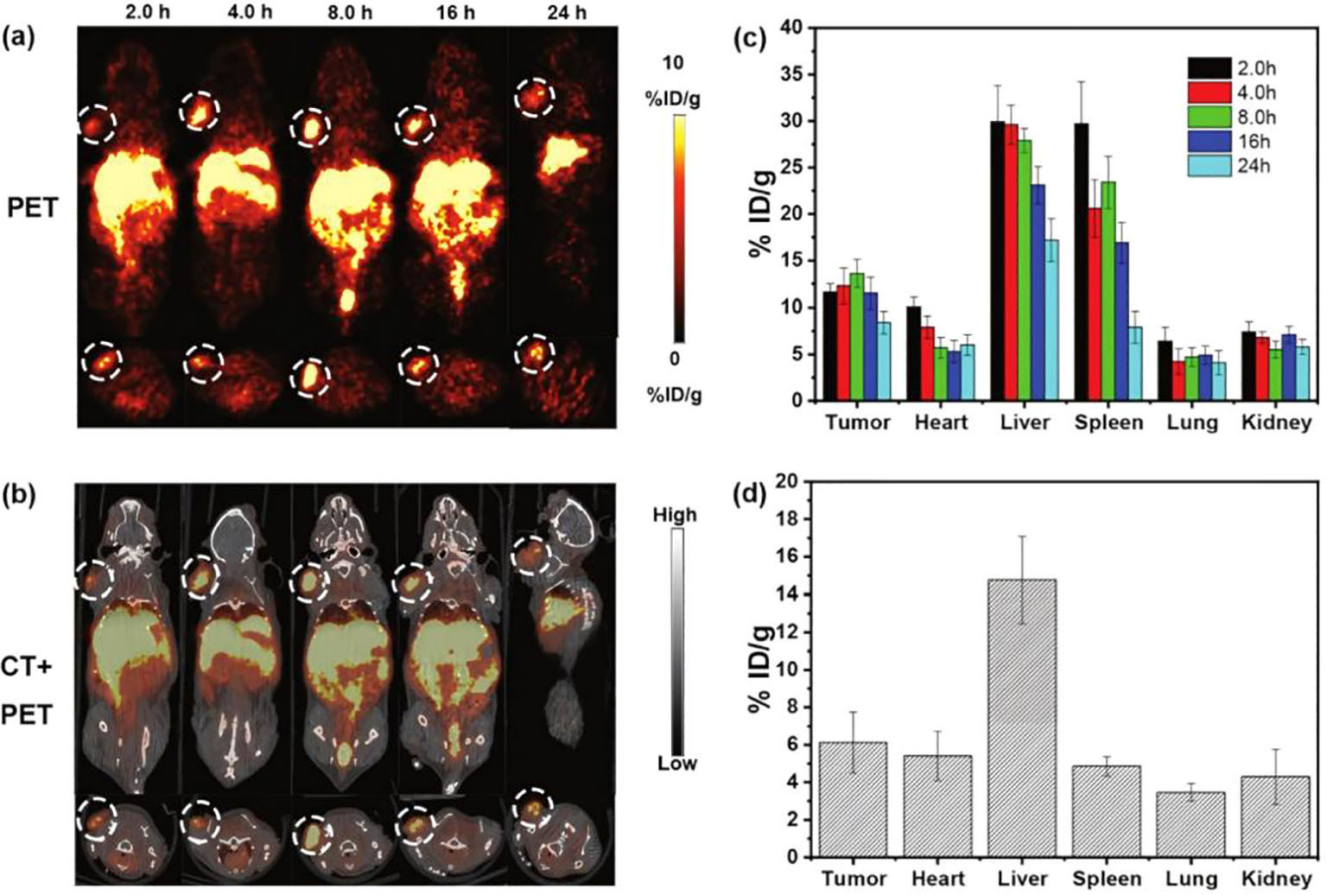
The representative decay-corrected whole-body PET/CT images of nude mice bearing A431 tumor (region circled by white dashed line) acquired at 2, 4, 8, 16, and 24 h postinjection of CH-4T/SLB–MSN–Mdot/^64^Cu^2+^, showing **(a)** the transaxial (bottom) and coronal (top) PET images and **(b)** the overlaid CT (gray) and PET (golden) images. **(c)** The region of interest (ROI)-calculated biodistribution of CH-4T/SLB–MSN–Mdot/^64^Cu^2+^ in mice at 2, 4, 8, 16, and 24 h postinjection. **(d)** Biodistribution of CH-4T/SLB–MSN–Mdot/^64^Cu^2+^ in mice at 25 h. Means ± SD, n = 3. ^©^ 2019 WILEY‐VCH Verlag GmbH & Co. KGaA, Weinheim.

Beyond dual-modality PET/fluorescence imaging, researchers are exploring integrating PET with second NIR-II fluorescence imaging. Zhang et al. engineered a hierarchical nanostructured hybrid platform that incorporates melanin quantum dots, mesoporous silica, the CH-4T dye, and the radionuclide ^64^Cu, enabling NIR-II fluorescence/PET dual-modality imaging (**Figures 7**, **8**) (108). This nanoprobe exhibits high fluorescence intensity, robust stability, and favorable biocompatibility, enabling tumor delineation and intraoperative resection guidance. It demonstrates potential in high-sensitivity preoperative diagnosis and precise image-guided surgery. This study presents a viable design strategy for the development of NTS1-targeted PET/NIR-II dual-modality molecular probes, which hold promise for further enhancing the imaging quality of preoperative tumor diagnosis and the accuracy of intraoperative navigation.

In addition, Cherenkov luminescence imaging (CLI), as an emerging nonfluorescent optical modality, is closely related to radionuclide imaging. In a clinical study, Pratt et al. showed that CLI exhibits acceptable patient-level concordance with standard nuclear medicine imaging and can provide valuable information to patients undergoing targeted radiotherapy (109). Boykoff and Grimm also noted that CLI holds substantial potential for intraoperative margin assessment (110). Although prior studies have not specifically evaluated CLI with NTS1-targeted radiotracers, CLI with any NTS1-targeted radiolabeled probe can serve as an adjunct optical modality, providing real-time, noninvasive intraoperative information and potentially improving the precision of tumor resection. A translational advantage of this approach is that it does not require additional fluorescent dyes, thereby avoiding dye-induced increases in molecular hydrophobicity and background signal. However, its signal intensity is constrained by radionuclide energy spectra and tissue optical properties, resulting in limited deep-tissue penetration, and its clinical implementation depends on specialized equipment and workflow integration. Therefore, CLI is better suited as a complementary imaging modality rather than a replacement.

In summary, multimodal probes combine the high sensitivity and quantitative capability of PET, the high spatial resolution and real-time imaging of fluorescence, and the deep tissue penetration of NIR-II, thereby providing a more comprehensive strategy for tumor diagnosis and intraoperative resection. However, designing and synthesizing multimodal probes is more complex and requires careful trade-offs among conjugation strategies for reporter moieties across different modalities, *in vivo* biodistribution, metabolic fate, and overall chemical and biological stability (111). Future research should explore novel combinations of imaging modalities and coupling architectures and optimize probe design with respect to chemical scaffolds, pharmacokinetic properties, and manufacturability, to enable more efficient and precise clinical translation for cancer diagnosis and therapy.

## 3 Applications of NTS1-targeted molecular probes in targeted tumor therapy

Targeted therapy is a cornerstone of modern oncology, aiming to suppress tumor growth by selectively engaging key molecular targets within tumor cells and their microenvironment while minimizing damage to normal tissues. NTS1 is highly expressed across multiple malignancies and therefore represents an attractive therapeutic target. NTS1-targeted molecular probes have been used primarily in radioligand therapy (RLT) and show strong potential for clinical translation.

### 3.1 Radioligand therapy

Radioligand therapy (RLT), including peptide receptor radionuclide therapy (PRRT), is a precision treatment strategy that delivers therapeutic radionuclides to tumor-associated molecular targets via peptides, antibodies, or small-molecule ligands (112–115). At present, β⁻-emitting radionuclides remain the most widely used therapeutic payloads. Among them, ^177^Lu is a representative radionuclide with a physical half-life of approximately 6.7 d, a moderate tissue penetration range, and accompanying γ-emission that supports post-therapy imaging and dosimetry, making it widely used in integrated diagnostic and therapeutic applications (116). In contrast, ^225^Ac is an α-emitting radionuclide with high linear energy transfer (LET), a physical half-life of approximately 10.0 d, a short path length, and potent cytotoxicity. It may offer particular advantages for eliminating micrometastatic lesions, but it also requires stricter control over targeting specificity and safety (115). Notably, in addition to its use in SPECT imaging, ^111^In is frequently applied in pre-therapeutic biodistribution and dosimetry assessment. Its potential therapeutic effects are mainly mediated by emitted Auger electrons and internal conversion electrons rather than by classical β⁻ radiation (117). For peptide-based RLT, the main targets are usually receptors expressed on the surface of tumor cells, although intracellular receptors and other intracellular targets may also have therapeutic potential when appropriate cell-permeable ligands are available (118, 119). In the case of NTS1, a cell-surface G protein-coupled receptor, it remains one of the most representative extracellular targets for peptide-based RLT (120). Preclinical studies have demonstrated promising therapeutic potential for NTS1-targeted radioligands. However, their translational value depends not only on receptor affinity but also on overall pharmacokinetics and dosimetry, including metabolic stability, tumor retention, tissue distribution, and dose burden to non-target organs (56, 57). Accordingly, recent research on NTS1-targeted RLT has shifted from a sole focus on improving receptor binding to a more systematic design approach that optimizes *in vivo* behavior while ensuring treatment safety.

#### 3.1.1 Targeted alpha therapy

Alpha-particle emitters, such as actinium-225 (^225^Ac), deliver high linear energy transfer (LET) over an extremely short range (on the order of tens of micrometers). These properties enable efficient tumor-cell killing while limiting damage to surrounding normal tissues, making them particularly suitable for treating micrometastases and isolated tumor cells (121, 122). Li et al. synthesized a di-DOTA-α,ϵ-Lys-NT (6–13) analog and radiolabeled it with ^225^Ac; therapeutic efficacy was evaluated in NTS1–positive PC3 and HT-29 xenograft mouse models. At doses of 18.5 and 37 kBq, [^225^Ac]di-DOTA-α,ϵ-Lys-NT (6–13) significantly prolonged survival, whereas systemic toxicity was observed at 74 kBq (55). These findings indicate that although NTS1-targeted molecular probes labeled with ^225^Ac are efficacious, precise dose optimization is required to minimize toxicity, a critical factor for clinical translation. Mahammad et al. demonstrated that the NTS1-targeted alpha-therapy agent [^225^Ac]FPI-2059 induced dose-dependent tumor suppression and prolonged survival in preclinical colorectal cancer models, supporting its clinical development for colorectal cancer (123). This study provides compelling evidence for NTS1-targeted alpha-particle radioligand therapy (alpha-RLT). By leveraging the high LET and short tissue range of alpha particles, this approach may offer theoretical benefits in settings where conventional radiotherapy is ineffective or where the tumor burden consists of numerous micrometastases. However, clinical translation still faces practical challenges, including the supply of α-emitting radionuclides, radiation safety, and manufacturing consistency. In addition, α-particle therapy is highly sensitive to organ dose constraints, making pre-therapeutic imaging-based patient selection and individualized dosimetry essential for maintaining an acceptable risk profile.

#### 3.1.2 Targeted beta therapy

Compared with α-emitting radionuclides, β⁻-emitting radionuclides have a longer tissue penetration range and are therefore more suitable for irradiating medium-to-large tumor lesions (124). In current studies of NTS1-targeted radioligand therapy, β⁻ emitters remain the predominant class. Among them, ^177^Lu has been the most extensively investigated because of its relatively well-established clinical use, moderate tissue distribution, and potential for integrated theranostic applications (116). Accordingly, existing studies have mainly focused on the *in vivo* distribution, antitumor activity, and translational clinical feasibility of ^177^Lu-labeled probes (56, 57).

In studies of NTS1-targeted molecular probes for tumor-directed therapy, early investigations mainly focused on characterizing the *in vivo* distribution of candidate ligands and assessing their potential for integrated diagnostic and therapeutic applications. While evaluating a series of non-peptidic NTS1 antagonists, Schulz et al. found that [¹¹¹In]In-3BP-227 showed favorable biodistribution and sustained tumor uptake, with peak tumor accumulation reaching 8.4 ± 3.1%ID/g at 6 h post-injection, thereby identifying it as a promising candidate for NTS1-targeted theranostic applications (100). The importance of this finding lies in showing that the 3BP-227 scaffold not only retains receptor-targeting capability but also exhibits favorable *in vivo* distribution and tumor retention, thus providing a basis for the subsequent development of therapeutic radioligands. Using the same ligand scaffold, Schulz et al. further evaluated the therapeutic potential of the ^177^Lu-labeled NTS1 antagonist [^177^Lu]Lu-3BP-227 in a rectal cancer xenograft mouse model. The results showed high tumor uptake (2.7 ± 1.6%ID/g at 69 h post-injection) and significant antitumor efficacy, with a tumor volume inhibition rate of 88%, without evidence of acute renal toxicity (57). These findings indicate that 3BP-227, as a radiolabeled NTS1 antagonist, not only shows robust *in vivo* retention but also demonstrates favorable preliminary safety and potential for clinical translation.

**Figure 9:**
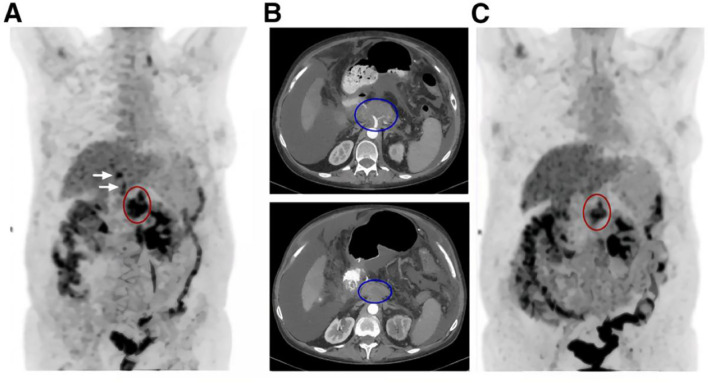
^18^F-FDG PET and CT scans of patient 3 before [**(A, B)** upper panel] and after [**(B)** lower panel, **(C)**] [^177^Lu]Lu-3BP-227 therapy. **(A)** ^18^FDG PET before [^177^Lu]Lu-3BP-227 therapy. Red oval: primary tumor; arrows: liver metastases. [**(B)** upper panel] Axial CT section; primary tumor (blue oval) before [^177^Lu]Lu-3BP-227 therapy. [**(B)**, lower panel] Axial CT section; primary tumor (blue oval) after 3 cycles of [^177^Lu]Lu-3BP-227 therapy. **(C)** ^18^FDG PET after 3 cycles of [^177^Lu]Lu-3BP-227 therapy. Red oval: primary tumor. This research was originally published in *JNM*. Richard P. Baum, Aviral Singh, Christiane Schuchardt, Harshad R. Kulkarni, Ingo Klette, Stefan Wiessalla, Frank Osterkamp, Ulrich Reineke and Christiane Smerling. 177Lu-3BP-227 for Neurotensin Receptor 1–Targeted Therapy of Metastatic Pancreatic Adenocarcinoma: First Clinical Results. J Nucl Med. 2018; 59:809-14. © SNMMI.

Building on this work, Baum et al. further reported preliminary clinical results for [^177^Lu]Lu-3BP-227 in patients with metastatic pancreatic ductal adenocarcinoma (PDAC). In one patient with ascites, treatment led to a marked reduction in both primary tumor size and metabolic activity (**Figure 9**), accompanied by a significant decrease in CA19–9 levels from 2555 U/mL to 220 U/mL and an overall survival of 11 months (56). This study suggests that NTS1-targeted radioligand therapy is feasible in advanced PDAC and provides a clinical basis for subsequent patient selection, individualized dosing, and cumulative dose management. However, the study was limited by the small sample size and patient heterogeneity, making it difficult to draw general conclusions regarding efficacy. Prospective studies are needed to identify the optimal patient population and to systematically assess the risks associated with cumulative radiation dose. As a representative antagonist-based therapeutic radioligand, the chemical structure of [^177^Lu]Lu-3BP-227 is shown in **Scheme 1D**.

Beyond radionuclide substitution, chemical structure-based optimization to enhance tumor retention has also emerged as an important strategy for improving the efficacy of NTS1-targeted β-particle therapy. Zhang et al. synthesized the NTS1-targeted conjugate [^177^Lu]Lu-NA-ET1, which incorporates a covalent cysteine protease inhibitor to enhance intratumoral retention and thereby increase intratumoral dose delivery in targeted radionuclide therapy (TRT). *In vitro*, [^177^Lu]Lu-NA-ET1 exhibited cellular uptake in NTS1-positive cell lines comparable to that of [^177^Lu]Lu-3BP-227, whereas *in vivo* biodistribution studies demonstrated higher tumor retention and greater radiation dose delivery. In mice, [^177^Lu]Lu-NA-ET1 was well tolerated. These findings suggest that a covalent inhibitor strategy can substantially improve tumor retention and dose delivery in TRT, potentially enhancing efficacy while reducing off-target toxicity (125). Chemical modification strategies that enhance intratumoral retention have the potential to improve the efficacy of radioligand therapy (RLT) while reducing radiation exposure to non-target organs.

The mechanisms of NTS1-targeted RLT extend beyond direct radionuclide-mediated cytotoxicity and may act synergistically with other treatment modalities. Valerie et al. reported that inhibiting NTS1 selectively sensitizes prostate cancer cells to ionizing radiation, promotes apoptosis, and reduces clonogenic survival. In prostate cancer models with high NTS1 expression, this radiosensitizing effect is observed irrespective of androgen dependence. The NTS1 antagonist SR48692 increases the radiosensitivity of PC-3M xenografts in mice by suppressing Epidermal growth factor receptor (EGFR) activation and downstream signaling pathways, thereby significantly reducing tumor burden (30). These findings suggest that, in addition to direct cytotoxic effects, NTS1-targeted strategies may serve as radiosensitizers when combined with external beam radiotherapy (EBRT), thereby potentially improving overall therapeutic efficacy.

Despite the considerable promise of NTS1-targeted radioligand therapy (RLT), its clinical translation and adoption remain challenging. Successful RLT depends on the precise delivery of radionuclides to tumor cells, maximizing tumor-absorbed dose while minimizing exposure to normal tissues. The ideal radionuclide pairs a half-life that aligns with biological kinetics with an appropriate radiation type, ensuring sufficient tumor-absorbed dose while keeping normal-tissue dose within acceptable limits to balance efficacy and toxicity (126). Further optimization of molecular probes, particularly their *in vivo* stability, biodistribution, and tumor penetration, is required, as their *in vivo* behavior directly determines the efficacy and safety of RLT. Particular attention should be paid to dose-limiting organs such as the kidneys and bone marrow. Renal uptake and tubular reabsorption may increase the cumulative radiation dose to the kidneys, whereas bone marrow suppression often limits the use of fractionated dosing and combination therapy. In addition, intra- and intertumoral heterogeneity can render certain cellular subpopulations insufficiently responsive to therapy, resulting in relapse or progression. This remains one of the principal bottlenecks in current RLT (127). The potential long-term toxicities of RLT, particularly renal and bone marrow effects (128, 129), require systematic clinical evaluation and the establishment of standardized follow-up protocols and dose-constraint guidelines. To address these challenges, future research should focus on optimizing probe design and dosing regimens, investigating synergistic combinations with chemotherapy, molecularly targeted therapies, or external-beam radiotherapy, and establishing safety and efficacy in prospective, phase-based clinical trials (130).

## 4 Theranostics

Theranostics integrates diagnostic and therapeutic functionalities into a single molecular probe, thereby enabling precision oncology (12). Theranostic probes enable clinicians to precisely localize lesions by molecular imaging, assess NTS1 expression, and predict treatment response; the same probe or its isotopic or chemical derivatives can then be used to deliver targeted therapy, with real-time monitoring of efficacy and adverse events (131–135). This “diagnose first, treat later, with continuous on-treatment evaluation” paradigm can substantially enhance treatment precision and efficiency (136, 137).

**Figure 10:**
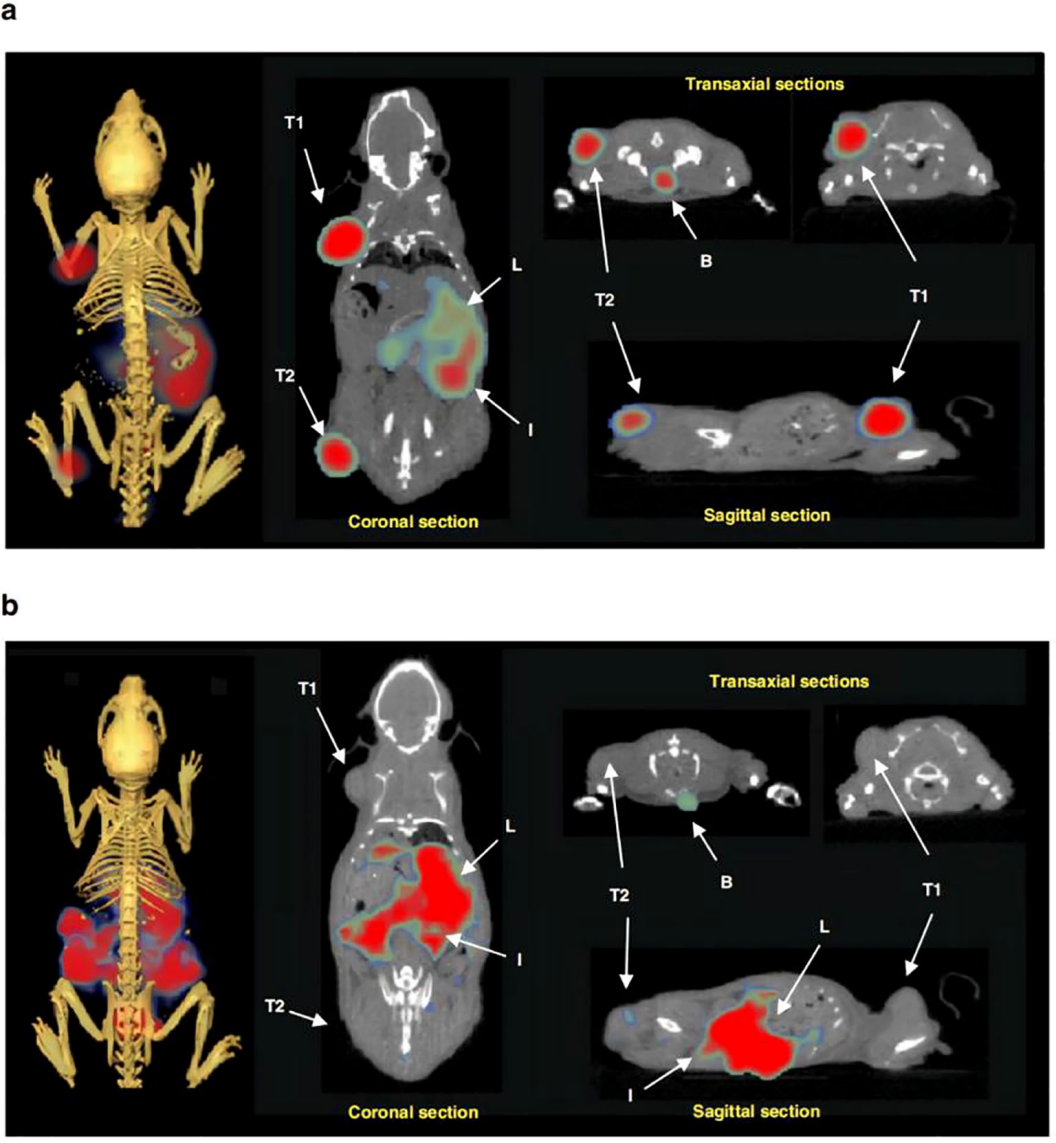
SPECT/CT images of mice bearing HT-29 tumour xenografts 1.5 h after i.v. injection of [^99m^Tc]Tc-NT-XIX (3.5–4 MBq). **(a)** Unblocked. **(b)** Blocked. Blocked animals received [^99m^Tc]Tc-NT-XIX co-injected with unlabelled NT-XI [18], 0.3 mg/mouse. T1, Upper tumour; T2, lower tumour; L, liver; I, intestines; B, bladder. Copyright © 2008, Springer-Verlag.

**Figure 11:**
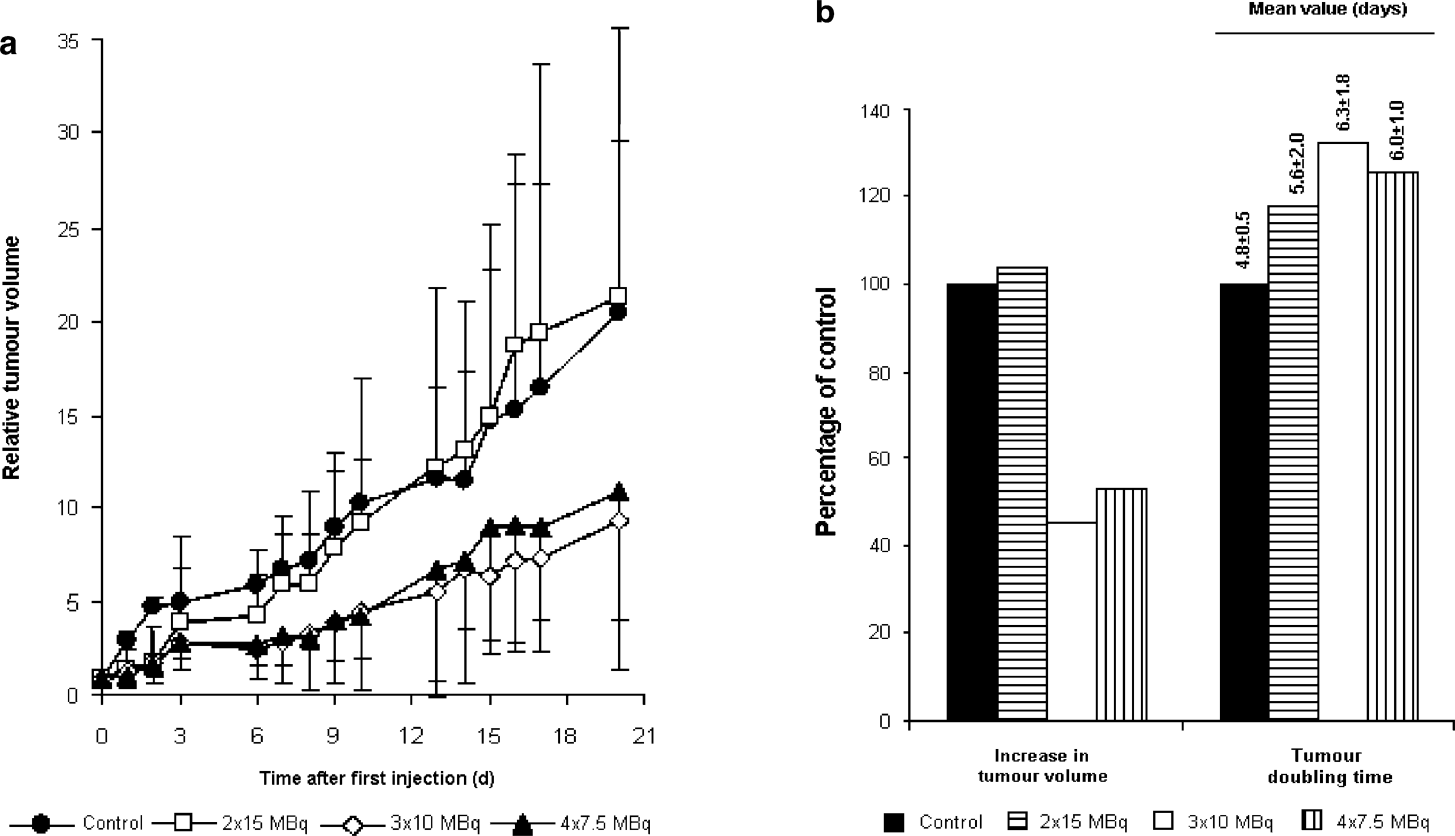
Therapeutic effect of [^188^Re]Re-NT-XIX (total accumulated dose 30 MBq/mouse) in mice with HT-29 tumour xenografts. **(a)** Timecourse tumour growth. Data are expressed as the volume of tumours relative to that in the same animals immediately before the first injection (mean ± SD of 4–7 animals). Statistical analysis (one-way ANOVA+Tukey’s *post hoc* test): p<0.05 all treated groups vs. control at days 1 and 2 after first injection; p<0.05 groups 3×10 and 4× 7.5 MBq vs. control at day 6 after first injection. **(b)** Comparison of tumour volume and tumour doubling time. Copyright ^©^ 2008, Springer-Verlag.

As a key tumor-associated receptor and biomarker, NTS1 has seen substantial progress in theranostic applications. Most NTS1-targeted radiotracers exhibit theranostic potential: paired constructs typically retain a common ligand scaffold, with different radionuclides selected for diagnosis or therapy, thereby closing the loop between imaging and treatment. Garayoa et al. used a triple-modification strategy to engineer the NT analog NT-XIX, which can be labeled with ^99m^Tc or ^188^Re for SPECT imaging and radioligand therapy, respectively (**Figure 10**). In HT-29 mouse xenografts, NT-XIX showed favorable tumor targeting and antitumor efficacy (**Figure 11**), providing initial proof of concept for NTS1-targeted theranostics (138).

Subsequently, Schulz et al. identified [^111^In]In-3BP-227 as a candidate theranostic ligand. In this system, ^111^In is primarily used for diagnostic SPECT imaging and pre-therapeutic *in vivo* biodistribution assessment, while the same ligand scaffold can be further labeled with therapeutic radionuclides to support subsequent therapeutic development (100). Li et al. synthesized di-DOTA NT derivatives, evaluated them for PET imaging with ^64^Cu or ^68^Ga, and subsequently administered ^225^Ac for therapy; this isotope-switching strategy delivers diagnostic imaging and treatment via radionuclide substitution, aligning with the theranostic paradigm (55). In addition, Lin et al. developed an NTS1-targeted probe, NOTA-NT-20.3, using ^55^Co for PET imaging and metastable ^58m^Co for radionuclide therapy, thereby integrating diagnostic imaging with radiotherapy in an HT-29 colon cancer model (139). Pharmacokinetic parameters derived from ^55^Co PET were used to guide ^58m^Co administration and dosimetry. The results demonstrated a moderate tumor response without significant toxicity, supporting the safety and feasibility of a theranostic approach to optimize radiation dosing and enable individualized treatment, and providing evidence for the utility of cobalt radionuclides in NTS1-targeted theranostics.

Multimodal imaging-guided theranostics further enhances therapeutic precision and procedural feasibility. Renard et al. designed a PET/fluorescence dual-modal imaging agent that supports PET-based evaluation and enables intraoperative fluorescence-guided resection, thereby markedly improving surgical accuracy for tumors requiring surgical intervention (19). Zhang et al. developed a PET/NIR-II dual-modal nanoprobe by integrating ^64^Cu with the CH-4T dye, enabling precise tumor diagnosis and image-guided surgery and offering a feasible pathway toward more complex theranostic strategies (108).

Theranostics enables patient selection, precise treatment, and real-time monitoring of therapeutic response, while partially mitigating challenges posed by tumor heterogeneity. Imaging-based assessment of NTS1 expression enables the identification of patients most likely to benefit from NTS1-targeted therapy, thereby avoiding ineffective treatment and advancing personalized medicine (140). Diagnostic imaging precisely localizes tumor lesions, guides the accurate delivery of therapeutics, and monitors treatment response in real time, enabling timely regimen adjustments (141). Incorporating multitarget strategies, such as dual-target probes directed against NTS1 and other tumor-associated targets, including prostate-specific membrane antigen (PSMA) and gastrin-releasing peptide receptor (GRPR), as explored by Previti et al. and Bodin et al., may help overcome tumor heterogeneity by targeting diverse cell populations, thereby broadening the effectiveness of both diagnostic and therapeutic applications (142–144). Despite substantial progress in NTS1 theranostics, challenges persist, including probe design complexity, optimizing dosimetry and toxicity, interference from the tumor microenvironment, and barriers to clinical translation (3, 55, 145). Future work may focus on developing next-generation theranostic probes (e.g., nanoplatforms integrating photodynamic therapy or immunotherapies) (146–148), advancing multitarget theranostics (149–151), engineering stimuli-responsive probes, and leveraging artificial intelligence and big data analytics to further advance precision and personalized medicine.

## 5 Current status and translational challenges of NTS1-targeted radiopharmaceuticals

Although NTS1-targeted radiopharmaceuticals have shown good receptor specificity and promising theranostic potential in preclinical studies, the available clinical evidence remains at an early stage. Current research mainly consists of small-scale human imaging studies, preliminary therapeutic investigations, and early-phase clinical trials, and the overall strength of the evidence remains limited. In contrast to targets such as SSTR and PSMA, for which relatively mature clinical pathways have been established, NTS1-targeted radiopharmaceuticals still lack sufficiently large, prospective, and multicenter studies to support standardized clinical application (52, 56, 152).

Existing studies indicate that NTS1-targeted probes exhibit some clinical translatability. Early SPECT studies provided preliminary evidence of tolerability in humans and initial imaging feasibility for these probes, whereas recent first-in-human PET studies suggest that NTS1-targeted PET imaging may further improve tumor contrast. At the same time, therapeutic probes labeled with radionuclides such as ^177^Lu have entered early-phase clinical development, suggesting that NTS1 may serve as an important target for integrated diagnostic and therapeutic strategies. Nevertheless, current findings remain largely limited to feasibility assessments and preliminary efficacy observations and are insufficient to define appropriate patient populations, predictive efficacy criteria, or standardized treatment protocols (42, 52, 56, 153–155). Representative human studies and early clinical development of NTS1-targeted radiopharmaceuticals are summarized in **Table 3**. The key challenges limiting the further clinical translation of NTS1-targeted radiopharmaceuticals mainly involve patient selection and the standardized assessment of receptor expression. Different studies have used diverse pathological assays, imaging metrics, and enrollment criteria, without a clear correspondence among these parameters, which partly compromises comparability across studies and limits clinical applicability. In addition, dosimetry optimization remains a central issue for therapeutic applications. In radioligand therapy, achieving a balance between maximizing the absorbed dose to tumors and minimizing radiation exposure to critical organs such as the kidneys and bone marrow is essential for both efficacy and safety. Furthermore, the *in vivo* stability, tumor retention, and non-target organ uptake of probes still require improvement. Without such optimization, even probes with high *in vitro* affinity may fail to translate into consistent and reliable clinical benefit (56, 157, 158).

**Table 3:** Representative human studies and early clinical development of NTS1-targeted radiopharmaceuticals.

Radiopharmaceutical	Clinical application	Tumor type	Clinical evidence/stage	Key point	Ref./Trial ID
[^99m^Tc]Tc-NT-XI	SPECT imaging	PDAC	Pilot human study	Safe biodistribution; tumor uptake demonstrated	(51)
[^68^Ga]Ga-NT-20.3	PET imaging	PDAC	First-in-human study	Well tolerated; human dosimetry established	(156)
[^68^Ga]Ga-DOTA-NT-20.3-Ibu	PET imaging	Lung cancer	Early human imaging study	Safe imaging; uptake correlated with NTS1 expression	(43)
[^68^Ga]Ga-DOTA-NT-20.3	PET imaging	PDAC	Registered prospective study	Safety and lesion detection under evaluation	NCT05048810
[^177^Lu]Lu-3BP-227	Radioligand therapy	Metastatic PDAC	Preliminary therapeutic study	Preliminary efficacy; kidney dose limitation	(56)
[^177^Lu]Lu-3BP-227	Radioligand therapy	NTS1-positive solid tumors	Phase I/II first-in-human study	terminated early during phase I dose escalation; phase II not initiated	EudraCT 2017-001263-20/NCT03525392
[¹¹¹In]In-FPI-2058/[^225^Ac]Ac-FPI-2059	Theranostic evaluation	NTS1-positive solid tumors	First-in-human Phase 1 trial	Imaging-guided selection; safety and dosimetry under evaluation	NCT05605522

PDAC, pancreatic ductal adenocarcinoma; NTS1, neurotensin receptor 1. “Clinical evidence/stage” refers to the current level of human evidence or trial phase. “Ref./Trial ID” denotes the corresponding reference or registered clinical trial identifier.

Beyond issues related to patient selection and the therapeutic window, the clinical advancement of NTS1-targeted radiopharmaceuticals is also constrained by regulatory requirements and practical implementation challenges. Because radiopharmaceuticals possess both pharmaceutical and radioactive properties, their development, production, and clinical use must comply with standards for pharmaceutical evaluation, quality management, and radiation safety. Once they enter clinical development, further translation depends on optimizing production processes, establishing quality control systems, implementing individualized dosimetry, and coordinating clinical trial design. This is particularly important for short-lived diagnostic and therapeutic radionuclides, for which stable synthesis, reproducible radiochemical quality, and cross-center consistency provide the basis for subsequent clinical studies and broader application. Overall, NTS1-targeted radiopharmaceuticals have gradually progressed from proof of concept to early-phase clinical development. However, the establishment of mature and generalizable clinical pathways will require higher-quality human studies, clearer patient stratification strategies, and more comprehensive dosimetry evaluation and implementation frameworks (152, 158–160).

## 6 Conclusions

In summary, NTS1 is overexpressed in multiple malignancies and provides a biological basis for both molecular imaging and radioligand therapy, making it a promising target for cancer diagnosis and treatment. Available evidence indicates that NTS1-targeted probes may have clinical value for lesion detection, staging, treatment response monitoring, intraoperative guidance, and theranostic management. In particular, PET/SPECT radiopharmaceuticals enable noninvasive evaluation of tumor burden and receptor expression, whereas multimodal probes may further bridge preoperative imaging and intraoperative guidance. Meanwhile, NTS1-targeted radioligands have shown encouraging antitumor activity in preclinical studies, and some antagonist-based probes have already entered early clinical evaluation. However, current evidence also suggests that the clinical value of these probes depends less on receptor affinity alone than on the overall balance among *in vivo* stability, circulation, and clearance behavior, tumor retention, non-target organ uptake, and radionuclide matching. This issue is particularly important for radioligand therapy, in which kidney and bone marrow dose, tumor heterogeneity, and long-term safety remain major factors limiting further clinical application. Although NTS1-targeted probes have progressed from proof of concept to early clinical translation, the available human evidence remains limited, and a well-established clinical pathway has not yet been achieved. Future progress will require continued efforts to address key challenges in clinical application, including further optimization of probe performance, more accurate patient selection and dosimetric evaluation, and improved manufacturing quality control and clinical validation. Meaningful advances in these areas will be essential for establishing NTS1-targeted molecular probes as more reliable tools for precision imaging and radioligand therapy in selected cancers.
