# Supplementary figures and images for: Advances in neurotensin receptor 1-targeted molecular probes for tumor molecular imaging and therapy
Source: Front Oncol. 2026 Apr 24;16:1817036. doi: 10.3389/fonc.2026.1817036 (PMC13152780; doi:10.3389/fonc.2026.1817036)

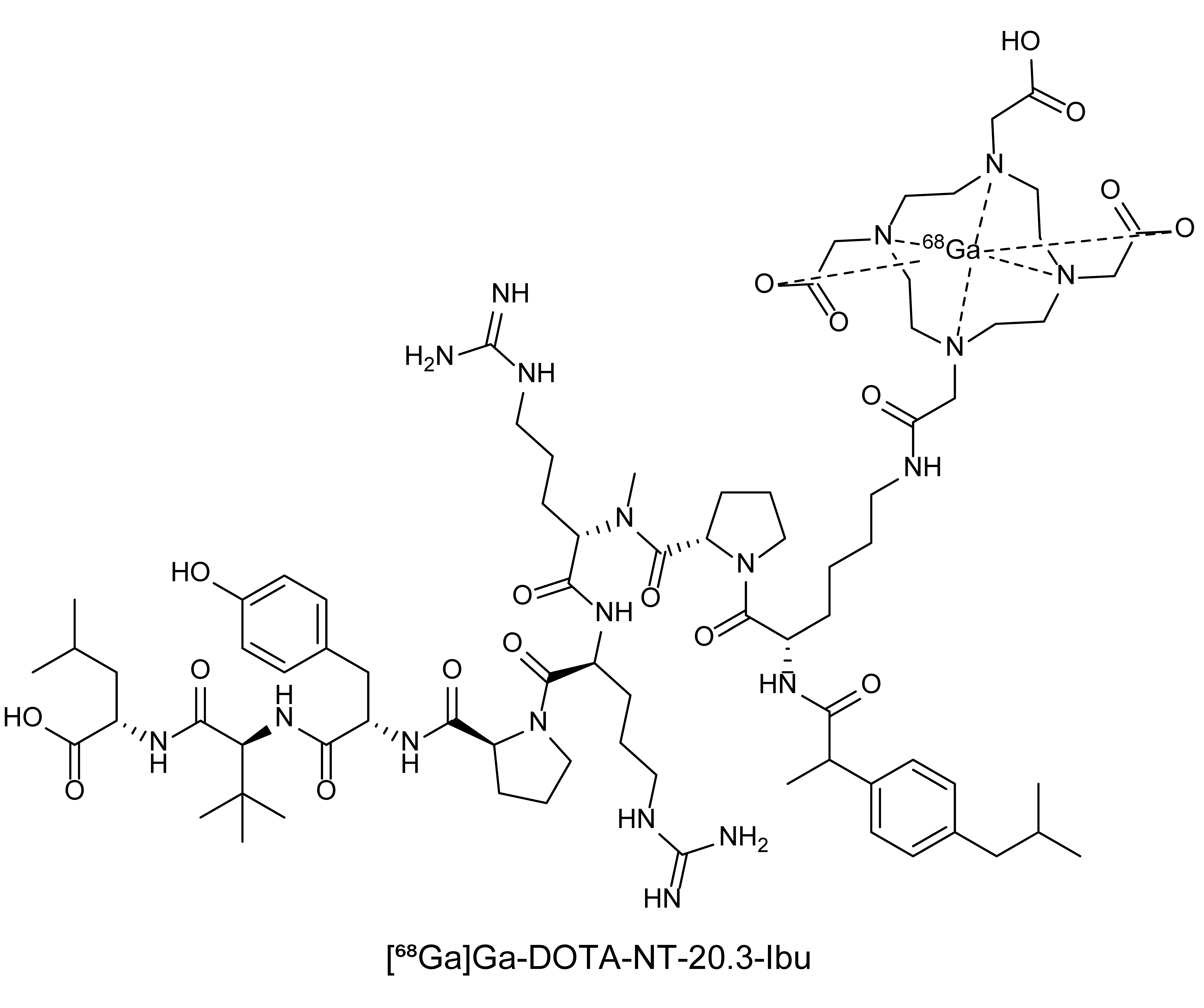

Supplement: Supplementary file 1 [file Image1.tif]

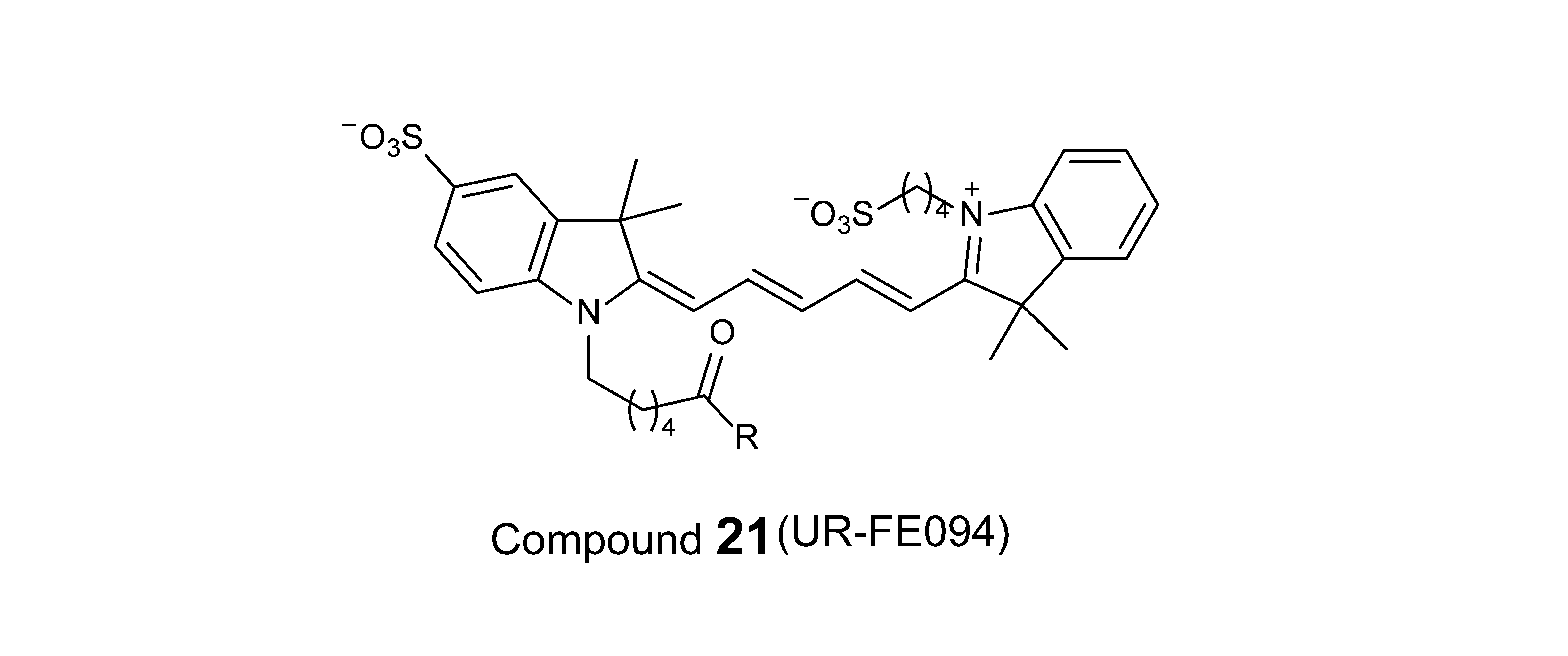

Supplement: Supplementary file 2 [file Image2.tif]
